# Leveraging Sarcopenia index by automated CT body composition analysis for pan cancer prognostic stratification

**DOI:** 10.1038/s41746-025-02016-z

**Published:** 2025-10-14

**Authors:** Katarzyna Borys, Johannes Haubold, Julius Keyl, Maria A. Bali, Riccardo De Angelis, Kévin Brou Boni, Nicolas Coquelet, Judith Kohnke, Giulia Baldini, Lennard Kroll, Sara Schramm, Andreas Stang, Eugen Malamutmann, Jens Kleesiek, Moon Kim, Stefan Kasper, Jens T. Siveke, Marcel Wiesweg, Anja Merkel-Jens, Benedikt M. Schaarschmidt, Viktor Gruenwald, Sebastian Bauer, Arzu Oezcelik, Servet Bölükbas, Ken Herrmann, Rainer Kimmig, Stephan Lang, Jürgen Treckmann, Martin Stuschke, Boris Hadaschik, Lale Umutlu, Michael Forsting, Dirk Schadendorf, Christoph M. Friedrich, Martin Schuler, René Hosch, Felix Nensa

**Affiliations:** 1 Institute of Diagnostic and Interventional Radiology and Neuroradiology, University Hospital Essen, Essen, Germany; 2 Institute for Artificial Intelligence in Medicine (IKIM), University Hospital Essen, Essen, Germany; 3 Department of Medical Oncology, West German Cancer Center, University Hospital Essen, University Duisburg-Essen, Essen, Germany; 4 Université Libre de Bruxelles (ULB), University Hospital Brussels, Institut Jules Bordet, Department of Radiology, Brussels, Belgium; 5 Université Libre de Bruxelles (ULB), Radiophysics and MRI Physics Laboratory, Brussels, Belgium; 6 Université Libre de Bruxelles (ULB), University Hospital Brussels, Institut Jules Bordet, Department of Medical Physics, Brussels, Belgium; 7 Department for Radiology and Nuclear Medicine, University Hospital Basel, Basel, Switzerland; 8 Institute for Medical Informatics, Biometry and Epidemiology (IMIBE), University Hospital Essen, Essen, Germany; 9 Department of General, Visceral and Transplantation Surgery, University Hospital Essen, Essen, Germany; 10 Bridge Institute of Experimental Tumor Therapy, West German Cancer Center, University Hospital Essen, Essen, Germany; 11 Division of Solid Tumor Translational Oncology, German Cancer Consortium (DKTK, Partner Site Essen) and German Cancer Research Center (DKFZ), Heidelberg, Germany; 12 Department of Thoracic Surgery, University Medicine Essen - Ruhrlandklinik, University Duisburg-Essen, Essen, Germany; 13 Department of Nuclear Medicine and German Cancer Consortium (DKTK), University Hospital Essen, University of Duisburg-Essen, Essen, Germany; 14 National Center for Tumor Diseases (NCT) West, Essen, Germany; 15 Department of Gynaecology and Obstetrics, West German Cancer Center, University Hospital Essen, Essen, Germany; 16 Department of Otorhinolaryngology, University Hospital Essen, University Hospital Duisburg-Essen, Essen, Germany; 17 Department of General, Visceral and Transplant Surgery, West German Cancer Center, University Hospital Essen (AöR), Essen, Germany; 18 Department of Radiotherapy, University Hospital Essen, University of Duisburg-Essen, Essen, Germany; 19 Department of Urology, Urological Oncology and Pediatric Urology, University of Duisburg-Essen, University Hospital Essen, Essen, Germany; 20 Department of Dermatology, West German Cancer Center, University Hospital Essen, University Duisburg-Essen, Essen, Germany; 21 Department of Computer Science, University of Applied Sciences and Arts Dortmund, Dortmund, Germany

**Keywords:** Cancer, Oncology

## Abstract

This study evaluates the CT-based volumetric sarcopenia index (SI) as a baseline prognostic factor for overall survival (OS) in 10,340 solid tumor patients (40% female). Automated body composition analysis was applied to internal baseline abdomen CTs and to thorax CTs. SI’s prognostic value was assessed using multivariable Cox proportional hazards regression, accelerated failure time models, and gradient-boosted machine learning. External validation included 439 patients (40% female). Higher SI was associated with prolonged OS in the internal abdomen (HR 0.56, 95% CI 0.52–0.59; P < 0.001) and thorax cohorts (HR 0.40, 95% CI 0.37–0.43; P < 0.001), as well as in the external validation cohort (HR 0.56, 95% CI 0.41–0.79; *P* < 0.001). Machine learning models identified SI as the most important factor in survival prediction. Our results demonstrate SI’s potential as a fully automated body composition feature for standard oncologic workflows.

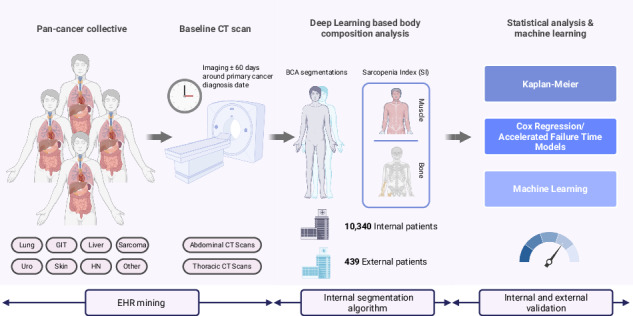

## Introduction

Body composition is increasingly recognized as a clinically relevant factor in oncology and beyond, supporting prognostication of clinical endpoints such as overall survival (OS), progression-free survival, treatment tolerance, and treatment response^1–6^. In this regard, Cachexia, a complex syndrome involving metabolic abnormalities and, in some cases, chronic inflammation, poses a considerable risk to cancer patients. Its adverse effects on body composition, including loss of muscle mass and altered adipose tissue distribution, negatively impact overall well-being and prognosis of patients^7–10^. It can compromise patients’ tolerance to chemotherapy, radiation therapy, and surgical interventions^6^. In this context, sarcopenia, which is defined as the progressive loss of skeletal muscle mass and function, emerged as a particularly relevant biomarker associated with poor clinical outcomes in cancer patients^11,12^. Fehrenbach et al. showed that reduced preoperative muscle mass is associated with lower OS in cancer patients, while Fleming et al. reported that patients with low skeletal muscle in colon cancer experience both poorer outcomes and increased pro-inflammatory activity^8,9^.

Despite this growing evidence, the methods used to perform body composition analysis (BCA) and assess prognostic factors such as sarcopenia remain limited in scope. Prior studies have commonly relied on conventional factors such as body mass index (BMI). Although BMI is easily obtainable and widely used in clinical settings, it does not directly reflect body composition, as it provides no information on the proportions of different tissues such as muscle, fat, or bone. Therefore, it only offers a very limited and indirect approximation. Nevertheless, BMI can serve as general indicator of underweight or obesity, respectively, and has been associated with OS in several cancers^10,13^. Moreover, studies on imaging-based BCA biomarkers applied non-volumetric segmentation approaches to quantify body composition. A common method involved the L3 area on a single slice of a CT scan, which historically emerged as the best approximator of whole-body composition when fully volumetric analysis was unfeasible^1,14,15^. Other methods, such as Dual-energy X-ray absorptiometry or bioelectrical impedance analysis, were also commonly used^7,16^. However, these anthropometric factors often provide an approximate representation of body composition and may not accurately reflect the distribution of important tissue compartments such as fat, muscle, and bone^7,10,11^. Alternative BCA biomarkers were explored to overcome these limitations^1,2,4,6,13,17–19^. Still, the prognostic value of volumetric CT-based BCA biomarkers in cancer patients on a large scale remains understudied. Moreover, evidence regarding the overall prognostic relevance of volumetric CT-based BCA biomarkers across different solid tumors is limited, particularly in large, unselected solid-tumor populations^20^. A substantial challenge in utilizing BCA biomarkers on a large scale is the time-consuming extraction process from CT or magnetic resonance imaging data, limiting scalability and feasibility in clinical routine^21–23^. Hence, there is a high need for automated and accurate BCA in clinical research and practice.

Recent advances in deep learning (DL) enabled a fully automated extraction of body composition features from medical imaging, offering a rapid and reproducible quantification of muscle, fat, and bone tissues across the entire scan. In response, volumetric BCA has gained traction as a scalable solution for CT scans^17,21^, which are acquired at primary staging in almost every cancer patient. This raises the question of whether routinely acquired staging CT scans, such as CT abdomen or CT thorax, can offer prognostic value regarding sarcopenia through automated volumetric BCA, particularly in the early phase after a primary cancer diagnosis, when patients may benefit most from timely physical or nutritional interventions.

In this work, we investigate the prognostic potential of a volumetric sarcopenia index (SI) as a non-invasive and widely available body composition feature, extracted from baseline standard CT images in a retrospective cohort of over 10,000 pan-cancer patients with solid tumors. The prognostic relevance of the SI was evaluated through uni- and multivariable survival analyses, supported by machine learning (ML) models using gradient-boosted survival trees. To assess generalizability, the SI’s predictive performance was further validated in an external cohort from a separate European academic center.

## Results

### Internal cohort’s patient characteristics

The internal abdomen cohort comprised 10,340 patients (40% female) treated at the West German Cancer Center in Essen, Germany. Median age was 64 years [interquartile range (IQR) 55–72] at the date of CT examination. As of 2022-02-16, 4295 (42%) patients were deceased. The date of primary cancer diagnosis ranged from July 2002 through December 2021, with a median follow-up time of 30 months [IQR 10–64]. CT scans were performed between August 2002 and January 2022, utilizing 88 scanner types from 6 manufacturers (Supplementary Table 1). A total of 1301 (13%) patients had a first treatment initiated with a median difference of 8 days [IQR 2–20] from the corresponding CT scan. For 7692 patients (74%), the M status was available, with 4584 (44%) having no distant metastasis (M0) and 3108 (30%) having at least one distant metastasis (M1). For the remaining 2648 (26%) patients, the M status was unknown (Mx). The cohort comprised 71 ICD-10 categories, with the most frequent code being lung cancer (C34, *N* = 3642, 35%). A complete list of the top 10 most frequent ICD-10 codes is listed in Supplementary Table 2. The median SI was higher in males (2.42 [IQR 2.12-2.75]) compared to females (2.24 [IQR 2.00–2.51]). Across M status groups, patients without distant metastasis (M0) had higher median SI values than those with metastasis (M1), particularly among females (M0: 2.30 vs. M1: 2.19). Table 1 shows the abdomen cohort’s baseline characteristics and the SI, divided by sex and M status (see Supplementary Table 3 for internal thorax cohort characteristics).

**Table 1:** Descriptive patient characteristics of the internal abdomen cohort

Characteristic	Female			Male		
Number of patients (%)	4105 (40)			6235 (60)		
Median age ([IQR] *in years*)	63 [54–71]			65 [56–72]		
Median survival time ([IQR] *in months*)	14 [5–34]			14 [5–32]		
Deceased patients as of 2022-02-16 (%)	1530 (37)			2765 (44)		
Gastrointestinal (%) [C15-C18, C20, C25]	600 (15)			890 (14)		
Head+Neck (%) [C01-C14, C32]	115 (3)			259 (4)		
Liver (%) [C22]	326 (8)			836 (8)		
Lung (%) [C34]	1482 (36)			2160 (35)		
Sarcoma (%) [C40, C41, C46, C49]	422 (10)			514 (8)		
Skin (%) [C43, C44]	261 (6)			363 (6)		
Urogenital (%) [C61, C62, C64, C67]	109 (3)			677 (11)		
Other (%) [Supplementary Table 5]	790 (19)			536 (9)		
Median Sarcopenia Index ([IQR])	2.24 [2.00–2.51]			2.42 [2.12–2.75]		
**M Status**	**M0**	**M1**	**Mx**	**M0**	**M1**	**Mx**
Total (%)	1693 (41)	1261 (31)	1151 (28)	2891 (46)	1847 (30)	1497 (24)
Median age ([IQR] *in years*)	63 [54-71]	62 [54–70]	62 [52–72]	65 [56–73]	63 [56–72]	65 [57–73]
Median survival time ([IQR] *in months*)	18 [7–40]	10 [4–24]	15 [6–41]	17 [7–38]	10 [4–21]	15 [5–39]
Deceased patients as of 2022-02-16 (%)	489 (29)	677 (54)	364 (32)	1018 (35)	1063 (58)	684 (46)
Gastrointestinal (%) [C15-C18, C20, C25]	172 (10)	260 (21)	168 (15)	333 (12)	348 (19)	209 (14)
Head+Neck (%) [C01-C14, C32]	85 (5)	8 (<1)	22 (2)	198 (7)	26 (1)	35 (2)
Liver (%) [C22]	47 (3)	78 (6)	201 (18)	140 (5)	140 (8)	556 (37)
Lung (%) [C34]	804 (48)	545 (43)	133 (12)	1202 (42)	794 (43)	164 (11)
Sarcoma (%) [C40, C41, C46, C49]	236 (14)	90 (7)	96 (8)	283 (10)	113 (6)	118 (8)
Skin (%) [C43, C44]	191 (11)	27 (2)	43 (4)	264 (9)	47 (3)	52 (4)
Urogenital (%) [C61, C62, C64, C67]	23 (2)	33 (3)	53 (5)	298 (10)	206 (11)	173 (12)
Other (%) [Supplementary Table 5]	135 (8)	220 (18)	435 (38)	173 (6)	173 (9)	190 (13)
Median Sarcopenia Index ([IQR])	2.30 [1.97–2.50]	2.19 [1.99–2.43]	2.32 [2.05–2.60]	2.43 [2.13–2.77]	2.38 [2.09–2.70]	2.44 [2.16–2.76]

The number of patients was indicated as total and percentages, as well as the number of deceased patients. In addition, other variables were displayed as median and IQR. Results are grouped by sex and M status (M0: non-metastatic, M1: metastatic, Mx: unknown).

### External validation cohort’s patient characteristics

A cohort of 439 patients (40% female) from the University Hospital Brussels was acquired for external validation. The median age was 66 years [IQR 57–74] at the CT examination date, and primary diagnosis dates ranged from October 2004 to October 2023, with a median follow-up time of 52 months [IQR 26–114]. Corresponding CT scans ranged from October 2004 to November 2023. Overall, 197 (45%) patients were deceased. For all patients, the M status was available, with 319 (73%) having no distant metastasis (M0) and 120 (27%) having at least one distant metastasis (M1). The cohort’s baseline characteristics and SI statistics are in Supplementary Table 4. The cohort represented six different cancer groups, with lung cancer (C34) being predominant.

### Longitudinal assessment of bone volume

The longitudinal stability of bone volume as a normalization factor for SI was assessed in a subcohort of 250 patients with paired baseline and longitudinal PET/CT scans. Across both the abdominal and thoracic body regions, bone volume remained largely stable over time, with small median differences and no significant changes in most subgroups, as provided in Table 2. The only exception was a statistically significant but minor decrease observed in the M0 subgroup of thorax (P = 0.029) with a median bone volume difference of −10.43 mL.

**Table 2:** Paired comparison of bone volume (mL) between 250 baseline and follow-up PET/CT scans across the abdominal and thoracic CT region, stratified by metastasis status (M0: non-metastatic, M1: metastatic, all: M0 + M1)

CT Region	M	N	Test	*P* value	Δ Median (mL)	IQR (mL)	Baseline (mL)	Follow-up (mL)
Abdomen	0	154	*t* test	0.280	+0.99	−75.44 to 49.25	2778.80	2811.53
	1	96	*t* test	0.321	−7.22	−55.48 to 41.73	2580.02	2535.28
	All	250	Wilcoxon	0.233	−1.69	−59.43 to 44.46	2714.49	2749.70
Thorax	0	154	*t* test	0.029	−10.43	−61.60 to 34.02	1820.92	1804.80
	1	96	Wilcoxon	0.571	+5.27	−45.93 to 57.76	1587.81	1567.95
	All	250	Wilcoxon	0.296	−4.31	−58.69 to 47.99	1731.93	1742.01

Median volume differences (Δ), interquartile ranges (IQR), and median baseline and follow-up volumes are reported. Statistical tests were selected based on normality distribution (paired *t* test if the normality assumption was fulfilled, otherwise Wilcoxon signed-rank test was used).

### Kaplan-Meier analysis for volumetric and non-volumetric BCA features

**Fig. 1:**
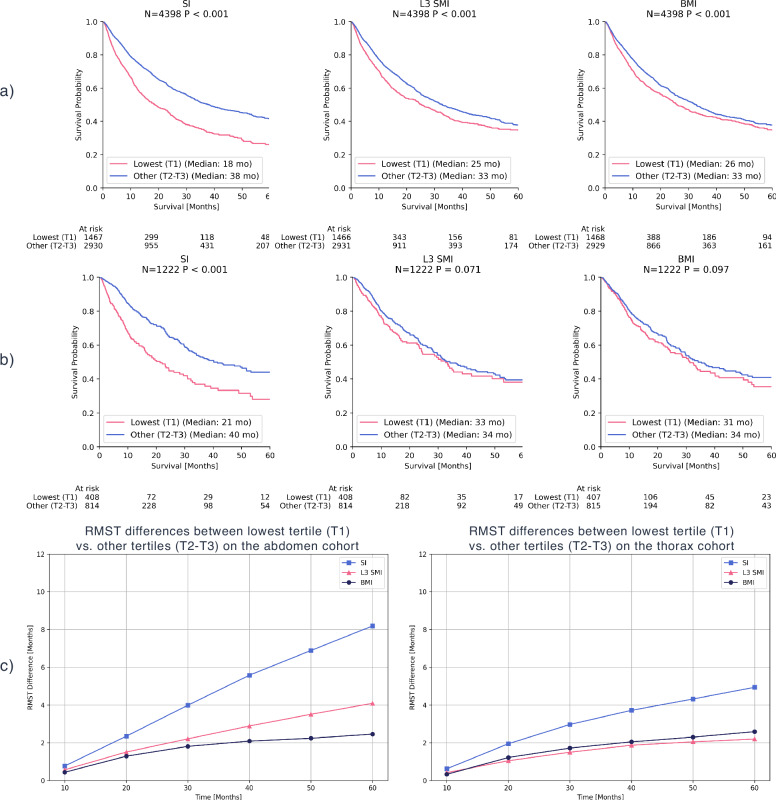
Kaplan-Meier survival curves and Restricted Mean Survival Time (RMST) differences for body composition features. Survival curves compare the lowest tertile (T1) with the combined upper tertiles (T2-T3) for the Sarcopenia Index (SI), Skeletal Muscle Index at the L3 vertebra (L3 SMI), and Body Mass Index (BMI) in **a** the internal abdomen cohort and **b** the internal thorax cohort. **c** Shows the corresponding differences in RMST between T1 and T2-T3 for all features across both CT regions.

Using Kaplan-Meier analysis, in both internal abdomen (N = 4398) and thorax (N = 1222) cohorts, the SI showed a superior survival separation between the lowest tertile (T1) and the combined upper tertiles (T2-T3), as presented in Fig. 1. Survival differences were statistically significant (P < 0.001) for SI, L3 SMI, and BMI in the abdomen cohort. Within the thorax cohort, L3 SMI and BMI showed non-significant separation (P = 0.071 and P = 0.097, respectively), while SI was significant (P < 0.001).

### Kaplan-Meier analysis across sex groups

**Fig. 2:**
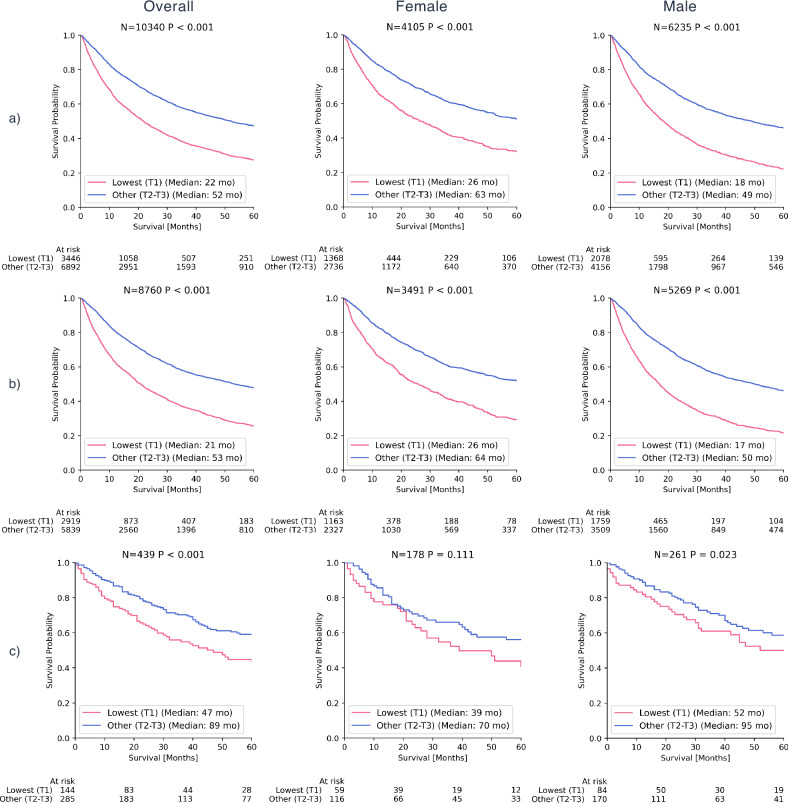
Kaplan-Meier survival curves by Sarcopenia Index (SI) tertiles, stratified by sex. Survival curves compare the lowest tertile (T1) with the combined upper tertiles (T2-T3) of the SI across three cohorts: **a** the internal abdomen cohort, **b** the internal thorax cohort, and **c** the external cohort.

Within the internal abdomen cohort, Kaplan-Meier analysis revealed a strong association between SI and OS across all patients, as well as within sex-specific subgroups (*P* < 0.001), as demonstrated in Fig. 2. These results were also replicated in the internal thorax cohort (*P* < 0.001). Within the external validation cohort, SI was associated with OS across all patients (*P* < 0.001) and for males (P = 0.023), whereas no significant association was observed for females (P = 0.111).

### Cox proportional hazards regression across cancer groups

In the Cox proportional hazards regression on the internal abdomen cohort data, a higher SI was significantly associated with prolonged OS with a HR of 0.56 (95% CI: 0.52-0.59, *P* < 0.001) as shown in Supplementary Fig. 1. This indicated that patients with higher SI values had a 44% lower risk of death compared to those with lower SI values. Similar results were observed in the internal thorax cohort, where higher SI values were associated with a 60% lower risk of death (HR of 0.40 (95% CI: 0.37–0.43, *P* < 0.001, see Supplementary Fig. 2). Lastly, in the external validation cohort, the association remained significant with an HR of 0.56 (95% CI: 0.41–0.79, *P* < 0.001). Stratified analysis by individual cancer groups was not performed in the external validation cohort due to limited sample sizes.

### Weibull accelerated failure time models across cancer groups

The Weibull AFT analysis of the internal abdomen cohort revealed that higher SI values were significantly associated with prolonged survival, corresponding to a time ratio of 2.69 (95% CI: 2.45–2.97, *P* < 0.001; Supplementary Fig. 3). This suggests that, on average, patients with higher SI experienced survival times nearly three times longer than those with lower SI. A similar association was observed in the internal thorax cohort, where higher SI values were linked to a more than fivefold increase in survival time (time ratio = 5.09, 95% CI: 4.42–5.87, *P* < 0.001; Supplementary Fig. 4). Consistently, across both cohorts, analyses within individual cancer groups also demonstrated significantly prolonged survival associated with higher SI values. In the external validation cohort, higher SI values were likewise associated with prolonged survival, corresponding to an approximate doubling of survival time (time ratio = 1.92, 95% CI: 1.30–2.82).

### Machine learning

**Fig. 3:**
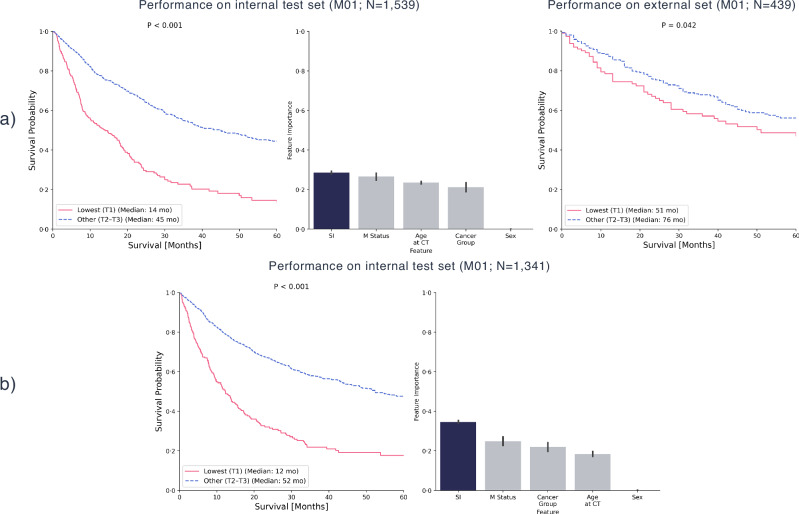
Machine learning results for combined metastatic status groups in the internal cohorts. Kaplan-Meier survival curves compared the lowest tertile (T1) with the combined upper tertiles (T2-T3) of survival times predicted by gradient-boosted survival trees. Results are shown for two models: **a** a model trained on the internal abdomen cohort, including both non-metastatic (M0) and metastatic (M1) patients, and **b** a model trained on the internal thorax cohort, also including both M0 and M1 patients. External validation was performed only for the abdomen-based model. Additionally, impurity-based feature importances are presented.

The M01 model trained on internal abdomen data achieved a mean TD-AUC of 0.74 [95% CI: 0.70–0.80], with a modest upward trend in TD-AUC over time (Supplementary Fig. 5). The mean bootstrapped C-index was 0.64 [95% CI: 0.61–0.68]. Kaplan-Meier survival curves showed a significant separation (P < 0.001) between the lowest tertile (T1) and the combined upper tertiles (T2-T3) of survival predictions. Among all features, the SI emerged as the most influential predictor. When applied to the external validation cohort, the Kaplan-Meier analysis showed a weaker but statistically significant separation (P = 0.042) of risk groups (Fig. 3). Comparable performance was observed for the M01 model trained on internal thorax data, achieving a TD-AUC of 0.72 [95% CI: 0.69–0.78] and a higher mean bootstrapped C-index of 0.68 [95% CI: 0.66–0.71]. Again, the SI was found to be the most important feature for prediction.

In subgroup-specific survival models stratified by M status (M0 and M1), the SI clearly separated survival outcomes across tertiles. While SI was the most important feature in M1 models, it remained a relevant but less dominant factor in M0 models, highlighting its prognostic value even when major clinical risk factors are accounted for (Supplementary Fig. 6).

Lastly, in ML models incorporating further body composition features, SI consistently outperformed both L3 SMI and BMI in survival discrimination, emerging as the most influential prognostic factor across both anatomical regions (Supplementary Fig. 7).

## Discussion

In this large-scale pan-cancer study, we investigated the prognostic value of the SI as a volumetric, non-invasive, and readily available body composition feature, extracted from baseline standard CT images in a retrospective cohort of over 10,000 cancer patients with solid tumors. Our results demonstrate that SI is a strong prognostic factor for OS, with lower SI values consistently associated with significantly worse outcomes across Cox regression, Kaplan-Meier analysis, Weibull AFT models, and ML models. The prognostic value of SI remained robust across the abdomen and thorax CT regions. In ML models stratified by M status, the SI continued to significantly separate survival outcomes across tertiles in both M0 and M1 groups. Notably, SI emerged as the most important predictive feature in M1 models, whereas it remained contributory, though not dominant, in M0 models. This suggests that SI may hold particular prognostic relevance in patients with more advanced disease stages, potentially reflecting the greater impact of muscle depletion in the context of systemic tumor burden (Supplementary Fig. 6). Recent literature increasingly supports the clinical relevance of volumetric BCA. For example, Bimurzayeva et al. demonstrated that 3D muscle and fat volumes extracted from abdominal CT scans outperformed conventional L3-based indices in predicting survival in colorectal cancer patients^24^. Similarly, Zheng et al. developed a CT-based body composition score incorporating volumetric assessments of muscle and fat, which significantly outperformed BMI and L3-SMI in predicting OS across digestive cancers^25^. Although our study focused solely on muscle and bone volumes, not including fat-based markers, our univariate Kaplan-Meier analysis comparing BMI, L3 SMI, and SI on a subcohort of internal patients aligns well with these findings. SI consistently outperformed BMI and L3 SMI in survival discrimination (Fig. 1, Supplementary Fig. 7) within both the abdomen and thorax cohorts. Mironchuk et al. further demonstrated that volumetric assessment of muscle and adipose tissue revealed nuanced, sex-specific survival associations across multiple solid tumor types in the TCGA cohort^26^. Notably, while slice-based measures at L3 failed to show sex-specific effects, volumetric analysis between L1 and L5 indicated that skeletal muscle was particularly associated with prolonged survival in females. They also reported that body composition metrics vary substantially by vertebral level and sex, highlighting a limitation of single-slice approaches. Also, Takahashi et al. reported that volumetric psoas muscle indices were superior prognostic markers than traditional cross-sectional area measures in elderly colorectal cancer patients^27^. These studies collectively reinforce a growing consensus that volumetric, AI-based BCA offers superior clinical utility compared to traditional anthropometric or slice-based measures. Our findings are consistent with this body of work. The SI addresses limitations of prior approaches and demonstrates robust prognostic value across a large-scale pan-cancer cohort. Notably, assessing the SI requires no additional imaging or manual segmentation, and as it is derived from standard CT scans, it facilitates seamless integration into clinical practice. The consistent prognostic performance of SI across anatomical regions, disease stages, and cancer groups, as well as in external validation, reinforces its clinical utility. Another notable approach of this study is the use of bone volume as a normalization reference, which was validated for longitudinal stability in a paired subcohort of 250 internal patients. This supports the hypothesis that the bone volume per body region can be used for normalization across timepoints using imaging data only. Importantly, this can be particularly valuable for retrospective analyses or scenarios, where patient height might not be available or is inconsistently documented. In addition, we also showed that the bone volume remains constant over multiple follow-ups for two specific patient cases with osteoblastic and osteolytic metastases, which were presented in Supplementary Figs. 8, 9. Although SI did not achieve statistical significance in female patients within the external validation cohort, the trend remained directionally consistent (Fig. 2). This discrepancy may relate to sample size limitations or sex-specific differences in muscle composition and warrants further investigation into sex-adjusted normalization strategies. Importantly, this study has several limitations. First, although the SI was externally validated, the ML models were trained exclusively on data from a single institution. Future multicenter training and harmonization of imaging protocols would enhance robustness. Second, lung cancer was overrepresented in the cohort, limiting disease-specific analyses for other tumor types. Future studies should ensure more balanced inclusion to enable stratified validation not only regarding cancer groups, but also across metastasis and sex groups. Third, due to the retrospective nature of the dataset, clinical covariates such as treatment regimens, comorbidities, and laboratory data were not available for multivariable analysis. Integrating these variables could further contextualize the prognostic value of SI. Also, due to sparse clinical documentation, it was not possible to determine cause-specific mortality. As a result, all-cause mortality was used within survival analyses. This approach may overestimate the association between SI and cancer-related outcomes, particularly in the absence of detailed comorbidity data. Future studies should aim to incorporate accurate cause-of-death information to enable cancer-specific survival analyses and better isolate the impact of the prognostic factor. Moreover, while Cox proportional hazards models were used in this analysis, we acknowledge potential violations of the proportional hazards assumption over long follow-up periods in heterogeneous real-world populations. To mitigate this, we employed AFT modeling as a complementary approach. Future work should explore time-varying covariate models to better capture dynamic and non-proportional effects. To reduce confounding and maintain biological consistency, we chose to limit our analysis to solid tumors and excluded malignancies with distinct behavior or cachexia patterns, such as hematologic, neuroendocrine, thyroid, and central nervous system tumors. This choice introduces limitations regarding the excluded entities, which require dedicated analyses in future studies. Lastly, this study focused solely on the muscle-based SI. Future research should evaluate the prognostic relevance of additional tissue compartments, including subcutaneous, visceral, and intramuscular adipose tissue, to capture broader body composition phenotypes. In conclusion, this study demonstrates that the SI, a fully automated and volumetric CT-derived body composition feature, is a prognostic factor for OS at baseline in patients with solid tumors. SI consistently outperforms BMI and L3-SMI, generalizes across anatomical regions and clinical subgroups, and has been externally validated in a pan-cancer cohort. Given its fully automated and straightforward acquisition, these findings suggest that incorporating SI into standard imaging-based staging routines could be a practical tool for risk stratification in oncological patients.

## Methods

The approval for this retrospective study was obtained by the Ethics Committee of the University Hospital Essen (approval number 21-10204-BO) and the Comité d’Ethique Institut Jules Bordet-Hopital Universitaire de Bruxelles (approval number CE 3771). Due to the study’s retrospective nature, the requirement of written informed consent was waived by the Ethics Committee. All data were fully anonymized before being included in the study.

### Internal cohort acquisition

Electronic health records from adult cancer patients treated at the University Hospital Essen were screened, totaling 57,620 records and referencing 59,3231 imaging studies. From these, 15,281 CT scans matched the inclusion criteria: Whole-body or abdominal CT scan, image acquisition within ±60 days of the first primary cancer diagnosis, slice thickness ≤5 mm, and reconstruction using a soft tissue kernel. Whole-body scans were included, as they could be selectively cropped to the anatomical region of interest. To minimize potential confounding and heterogeneity, we excluded patients with lympho-hematologic malignancies, neuroendocrine tumors, thyroid cancers, and primary brain tumors based on ICD-10-GM codes^28^. Hematologic malignancies often lack discrete tumor masses and show inconsistent associations with sarcopenia and prognosis^29^. Well-differentiated neuroendocrine tumors are frequently indolent and may remain undiagnosed for prolonged periods^30^. In thyroid cancer, limited systemic involvement at diagnosis and good survival prognoses may distort the relevance of body composition^31^. For brain tumors, early neurologic symptoms and immobility can impact muscle mass irrespective of cachexia^32^.

Following these criteria, 11,086 patients were eligible. Lastly, imaging studies having a mismatch between DICOM metadata and the scanned body region (which was detected via our Body and Organ Analysis algorithm^33^) were further excluded. For patients with multiple eligible CT scans, only the scan closest to the time of their primary cancer diagnosis was used. Scans from relapsed patients were excluded to ensure that each patient was represented only once. This resulted in a total number of 10,340 distinct patients being eligible for analysis.

If available, a baseline CT thorax scan was also extracted for patients already included in the abdomen cohort, creating a subcohort for analyses between the thoracic and abdominal CT regions. This approach ensured representation of the two most common staging protocols. Importantly, the thorax subcohort did not comprise a separate patient group. Instead, it was restricted to individuals already included in the abdomen cohort. Consequently, each patient contributed a single CT scan per anatomical region. Among the 10,340 patients having an abdominal scan, 8760 (85%) also had a baseline thorax CT scan following the same inclusion criteria. Using these two cohorts, the prognostic value of the SI between the abdominal and thoracic regions could be compared among internally acquired patients.

**Fig. 4:**
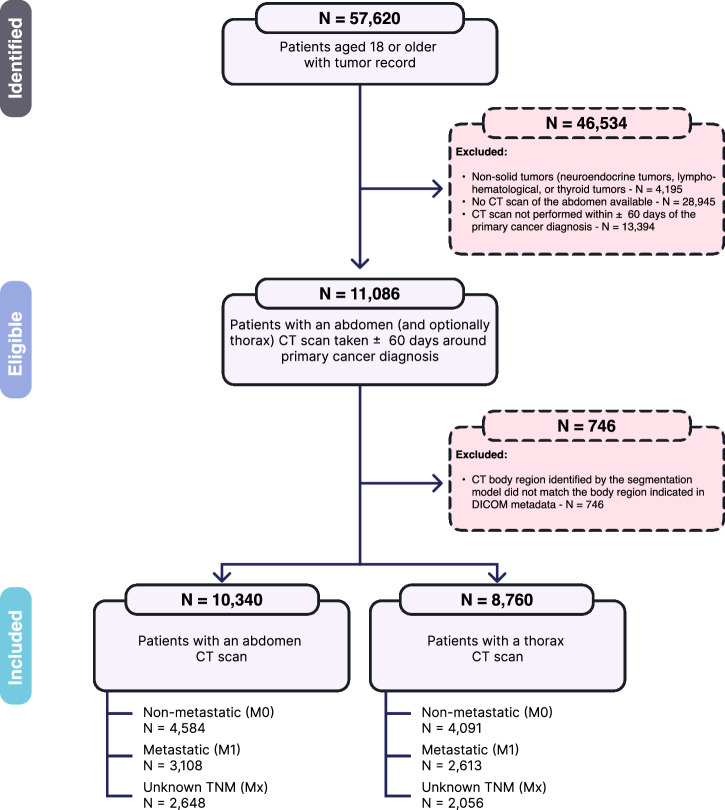
Flowchart of cohort selection. From an initial pool of 57,620 patients, 11,086 remained after excluding those without eligible CT scans and non-target tumor types. Further exclusion of 746 patients based on mismatched series metadata and scanned body regions yielded a final abdominal CT cohort of 10,340 patients. Additionally, a subcohort of 8760 patients with thorax CT scans was identified. Patients were classified according to their metastatic status into M0 (non-metastatic), M1 (presence of distant metastases), and Mx (staging information unavailable within ±60 days of primary cancer diagnosis).

In addition to CT imaging, baseline clinical variables, including sex, age at CT, cancer group, and metastatic status (M status), were acquired. The cancer groups were formed by combining multiple ICD-10 codes for primary cancer diagnoses into broader categories: Gastrointestinal (C15-C18, C20, C25), Head+Neck (C01-C14, C32), Liver (C22), Lung (C34), Sarcoma (C40, C41, C46, C49), Skin (C43, C44), and Urogenital (C61, C62, C64, C67). Additionally, 29 less common ICD codes within our cohort were grouped into the “other” category (see Supplementary Table 5) to reduce sparsity and ensure that these patients were included in the analysis. The M status was derived from the clinical TNM staging, which was assessed within a ± 60-day window around the primary cancer diagnosis. Patients were classified as non-metastatic (M0: no distant metastasis), metastatic (M1: at least one distant metastasis), or unknown (Mx: no staging available). Within analyses, SI and age at CT were treated as continuous variables, while sex, cancer group, and M status were dichotomized. A diagram visualizing the cohort selection process is presented in Fig. 4.

### External cohort acquisition

With adherence to identical inclusion criteria as those applied for the internal cohort, an external validation cohort was compiled at the University Hospital Brussels. This cohort included eligible patients with solid tumor records who underwent baseline CT abdominal scans. The primary purpose of the external validation cohort was to assess whether the results obtained from the internal cohort could be replicated in an independent external cohort, strengthening the SI’s generalizability.

### CT segmentation algorithm

Body composition analysis was performed using a pre-trained DL-based segmentation network called Body and Organ Analysis (BOA)^33^. The BOA enabled an automated segmentation and volume quantification (mL) of several tissues from CT scans. Importantly, this builds on a dual process: First, the BOA detects relevant body regions within a CT (e.g., abdominal cavity, thoracic cavity, and mediastinum; see Supplementary Figs. 10–12 and Supplementary Table 6). In the second step, tissues such as muscle, bone, visceral adipose tissue, subcutaneous adipose tissue, and others are segmented and quantified as volumes within the previously detected body regions^33^. In this work, we specifically focused on the abdominal and thoracic cavities as regions of interest to perform the analyses on consistent and clinically relevant CT areas.

### Longitudinal assessment of bone volume

To account for variations in body size between individuals, muscle volume was normalized by bone volume in the respective body region. Bone volume was selected as a fully image-derived, automatically extractable parameter from CT imaging, serving as a practical alternative to patient height, which is a commonly used normalization factor in body composition studies^14,18^, but oftentimes unavailable or unreliable in retrospective clinical datasets. We reason that bone volume may serve as a normalization alternative, even in patients with osteoblastic or osteolytic metastases (Supplementary Figs. 8, 9). To assess the stability of bone volume over time, we conducted a longitudinal analysis in a subset of patients with both baseline and follow-up PET/CT scans acquired between 90 and 365 days apart, ensuring sufficient time for potential skeletal changes to manifest. Bone volume differences (mL) were computed per patient for the abdominal and thoracic regions. Normality of the paired differences was assessed using a Shapiro-Wilk test. Depending on the distribution, either a paired t-test or a Wilcoxon signed-rank test was applied to evaluate whether the differences in bone volume over time were statistically significant overall and across metastatic statuses (M0, M1).

### Calculation of Sarcopenia index

After segmentation and quantification of bone and muscle volumes, the SI was computed as the ratio of muscle volume (mL) to bone volume (mL) in the respective body region.

1 $$Sarcopenia\,Index\,(SI)=\frac{Muscle\,(mL)}{Bone\,(mL)}$$

The resulting SI is a continuous variable, where the smallest SI value represents a scenario where muscle volume is nearly absent, potentially in cases of extreme muscle wasting, such as in cachexia^34^. A large SI value occurs when muscle volume is maximized relative to bone volume. While the upper limits of SI may vary, values greater than 1.0 generally indicate that muscle mass is higher relative to bone volume.

### Volumetric versus non-volumetric BCA

To further evaluate the prognostic utility of the proposed volumetric SI, we compared it against established non-volumetric body composition features in a subset of internal patients for whom height and BMI data were available. Specifically, we evaluated OS associations for the features SI, L3 Skeletal Muscle Index (L3 SMI), and BMI:

2 $$Body\,Mass\,Index\,(BMI)=\frac{Weight\,(kg)}{Height\,{(m)}^{2}}$$

3 $$Skeletal\,Muscle\,Index\,at\,L3\,(L3\,SMI)=\frac{Skeletal\,Muscle\,area\,at\,L3\,(c{m}^{2})}{Height\,{(m)}^{2}}$$

The skeletal muscle area was derived from the axial CT slice located at the midpoint of the L3 vertebra. For each feature, patients were stratified by tertiles, and survival differences were assessed by comparing the lowest tertile (T1) against the combined upper tertiles (T2-T3) using Kaplan-Meier analysis and the log-rank test. In addition, we quantified differences in Restricted Mean Survival Time (RMST) between the resulting risk groups.

### Statistical analysis

The two outcome variables used in survival modeling were time-to-event, defined as survival time in months from primary cancer diagnosis to death of any cause or censoring, and event status, a binary variable indicating whether death occurred (1 = deceased, 0 = censored). The cut-off date for follow-up was 2022-02-16. Patients alive at the cut-off date were right-censored at their date of last contact. All analyses were conducted separately on the internal abdomen and thorax cohorts. The external cohort, which consisted exclusively of abdominal CT scans, was used for validation.

The association between SI and OS was first assessed using the Kaplan-Meier method with comparison via the log-rank test. Patients were stratified into tertiles based on SI values, and survival curves were compared between the lowest tertile (T1) and the combined upper tertiles (T2-T3). Statistical significance was set at an alpha level of P = 0.05. To estimate hazard ratios (HRs) and 95% confidence intervals (CIs) for SI, a multivariate Cox proportional hazards regression was employed, which was adjusted for baseline clinical factors, including age at CT, sex, cancer type, and M status.

Because several covariates, most notably SI, violated the proportional hazards assumption, we fitted Cox models using robust standard errors (Huber sandwich estimator^35^). This approach allows for consistent estimation of standard errors under misspecification of the hazard function and enables interpretation of resulting HRs as general, time-averaged effects. To complement the Cox models and relax the proportional hazards assumption, we employed Weibull accelerated failure time (AFT)^36^ models. AFT models describe whether covariates accelerate or decelerate event timing and offer an alternative when proportionality cannot be assumed. These models were also adjusted for relevant clinical covariates.

Finally, to evaluate the predictive utility of SI in a multivariable, non-parametric framework, we trained gradient-boosted survival trees (GBST)^37^ using the inverse probability of censoring weighting least squares loss function. Given the prognostic importance of M status, separate models were developed: one combining both metastatic and non-metastatic patients (M01), one restricted to non-metastatic patients (M0), and one restricted to metastatic patients (M1). This stratified modeling approach enabled the assessment of SI’s performance and relevance within distinct clinical subgroups defined by M status. All models were trained with 80% of each cohort’s dataset. The train-test split was performed using stratified sampling by event status (1: deceased vs. 0: not deceased) and a fixed random seed to ensure a reproducible balance between censored and uncensored observations. To mitigate overfitting and enhance generalizability, a fivefold cross-validation (CV) was implemented within the training. Notably, each patient was included only once, either as a training or test sample, to avoid data leakage. Out-of-fold predictions were used to assign patients to tertiles of predicted survival, which stratified the held-out test data. Survival curves were estimated using the Kaplan-Meier method. As in prior analyses, patients in the lowest tertiles (T1) were compared to those in the combined upper tertiles (T2-T3) using the log-rank test. Feature importance was assessed using impurity-based measures computed during model training and aggregated across CV-folds. Model performance was evaluated using Harrell’s concordance index (C-index)^38^ and time-dependent area under the receiver-operating characteristic curve (TD-AUC)^39^, which were both computed on the test set. CIs for these metrics were calculated using 1000 bootstrap resamples with a fixed random state. To assess the generalizability of the GBST models, we validated them using the external cohort. Patients in the external cohort were assigned to survival risk tertiles based on the prediction thresholds (tertiles) derived from the internal model’s out-of-fold predictions. Kaplan-Meier survival curves were generated for these risk groups, mirroring the internal validation procedure.

**Fig. 5:**
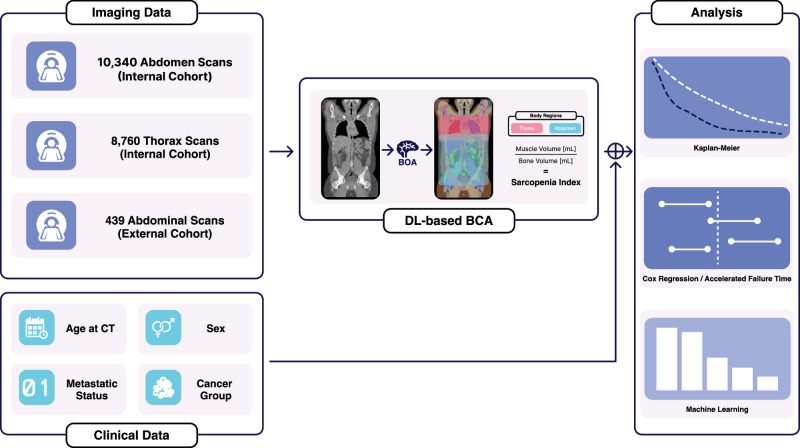
Schematic overview of the study design and analysis. The internal cohorts include baseline CT scans of both the abdomen and thorax, while the external validation cohort comprises abdominal CT scans only. All cohorts underwent automated body composition analysis (BCA) to extract the Sarcopenia Index (SI). Subsequent statistical and machine learning analyses were performed to evaluate the prognostic value of SI for overall survival.

Analyses were performed using Python 3.10 and the packages lifelines^40^, SciKit^41^, and Scikit-Survival^37^. An overview of the study design and analysis is visualized in Fig. 5.

## Supplementary information

Supplementary Information

STROBE_checklist

## Data Availability

The dataset used in this study is not publicly available. Individuals or academic organizations interested in utilizing this dataset must submit a detailed request to [[Data-Governance@uk-essen.de](mailto:Data-Governance@uk-essen.de)], which will be reviewed on a case-by-case basis.
