# Supplementary material for: Leveraging Sarcopenia index by automated CT body composition analysis for pan cancer prognostic stratification
Source: NPJ Digit Med. 2025 Oct 14;8:611. doi: 10.1038/s41746-025-02016-z (PMC12521393; doi:10.1038/s41746-025-02016-z)
Supplement: Supplementary file 1 — Supplementary Information [file 41746_2025_2016_MOESM1_ESM.pdf]

Leveraging Sarcopenia Index by automated CT body composition analysis for pan cancer prognostic stratification

Supplementary Information:

Supplementary Table 1: Overview of CT scanners used for image acquisition in the internal cohort

Supplementary Table 2: Top 10 ICD-10 codes in the internal cohort

Supplementary Table 3: Patient characteristics of the internal thorax cohort

Supplementary Table 4: Patient characteristics of the external validation cohort

Supplementary Table 5: ICD-10 codes included in the cancer group "Other"

Supplementary Table 6: Processing times of the body composition analysis (BCA) network across different CT scan types

Supplementary Figure 1: Effect of the Sarcopenia Index from multivariable Cox regression in the internal abdomen cohort

Supplementary Figure 2: Effect of the Sarcopenia Index from multivariable Cox regression in the internal thorax cohort

Supplementary Figure 3: Effect of the Sarcopenia Index from multivariable Accelerated Failure Time (AFT) model in the internal abdomen cohort

Supplementary Figure 4: Effect of the Sarcopenia Index from multivariable Accelerated Failure Time (AFT) model in the internal thorax cohort

Supplementary Figure 5: Time-dependent AUC for internal abdomen and thorax models with non-metastatic and metastatic patients (M0 and M1)

Supplementary Figure 6: Machine learning results for distinct metastasis groups based on the internal abdomen and thorax cohorts for non-metastatic (M0) and metastatic (M1) models

Supplementary Figure 7: Machine learning results for SI, L3-SMI, and BMI comparison

Supplementary Figure 8: Longitudinal body composition changes in a lung cancer patient with osteoblastic metastasis

Supplementary Figure 9: Longitudinal body composition changes in a patient with osteolytic metastasis

Supplementary Figure 10: Example report from the body composition analysis (BCA) network for a whole-body CT scan

Supplementary Figure 11: Tissue segmentation examples from the body composition analysis (BCA) network

Supplementary Figure 12: Feature extraction using standardized body regions in body composition analysis (BCA)

Manufacturer	Scanner
Canon Medical Systems	Aquilion Prime SP
GE Medical Systems	BrightSpeed<br>LightSpeed16<br>Optima CT540<br>Optima CT660<br>Discovery CT750 HD<br>Optima CT520 Series<br>Revolution EVO<br>LightSpeed Ultra<br>HiSpeed QX/i<br>Revolution HD<br>BrightSpeed S<br>Optima CT580<br>Revolution CT<br>Brivo CT385 Series<br>Discovery ST
Philips	Brilliance 16<br>Brilliance Big Bore<br>Ingenuity CT<br>Ingenuity TF PET/CT<br>Ingenuity Flex<br>Brilliance 64<br>Brilliance 6<br>Brilliance 40<br>iCT 256<br>Ingenuity Core<br>Big Bore<br>Ingenuity Core 128<br>Access CT<br>GeminiGXL 16<br>GEMINI TF Big Bore<br>GeminiGXL 10<br>Philips CT Aura<br>Incisive CT<br>GEMINI TF TOF 16<br>Brilliance 6 Dunlee<br>Mx8000 IDT 16
PNMS	MX 16-Slice
Siemens	Biograph128<br>SOMATOM Force<br>SOMATOM Definition Edge<br>SOMATOM Definition AS<br>SOMATOM Definition Flash<br>SOMATOM Definition AS+<br>Volume Zoom<br>SOMATOM Scope<br>Emotion 16 (2010)<br>Sensation 16<br>Emotion Duo<br>Biograph64<br>Sensation Open<br>Sensation 4<br>Emotion 16 (2007)<br>Emotion 16<br>Definition<br>SOMATOM Definition<br>Biograph 6<br>Biograph64_Vision 600<br>Emotion 6 (2007)<br>Sensation 64<br>Biograph20<br>Emotion 6<br>SOMATOM Perspective<br>SOMATOM X.cite<br>SOMATOM go.Up<br>Biograph 128 Edge<br>Sensation 40

	Biograph 16<br>Perspective<br>Biograph 128<br>SOMATOM go.All<br>Sensation Cardiac 64<br>Spirit<br>Biograph128Edge<br>Symbia Intevo 2<br>Biograph 64<br>Scope<br>Symbia T2<br>Biograph Horizon<br>Symbia Intevo 16<br>Biograph 40<br>SOMATOM Edge Plus<br>Definition AS<br>SOMATOM Drive
Toshiba	Aquilion<br>Aquilion Lightning<br>Aquilion ONE<br>Aquilion PRIME

Supplementary Table 1. Overview of CT scanners used for image acquisition in the internal cohort: This table provides a summary of all CT scanner models that were used to acquire imaging data within the internal imaging cohort. The overview includes manufacturer names and scanner models.

ICD-10 code	ICD-Description	Total (Female %)	Age (median [IQR])
C34	Malignant neoplasm of bronchus and lung	3642 (41)	68 [14]
C22	Malignant neoplasm of liver and intrahepatic bile ducts	964 (26)	69 [14]
C49	Malignant neoplasm of other connective and soft tissues	762 (46)	60 [22]
C43	Malignant melanoma of skin	467 (43)	66 [23]
C25	Malignant neoplasm of pancreas	360 (46)	65 [16]
C18	Malignant neoplasm of colon	286 (49)	68 [19]
C80	Malignant neoplasm without specification of site	263 (44)	65 [15]
C50	Malignant neoplasm of breast	261 (98)	59 [21]
C16	Malignant neoplasm of stomach	260 (44)	64 [19]
C61	Malignant neoplasm of prostate	258 (0)	72 [13]

Supplementary Table 2. Top 10 ICD-10 codes in the internal cohort: The table lists the ten most frequent ICD-10-GM diagnosis codes observed within the internal cohort, presented in descending order based on prevalence. The distribution highlights the dominant diagnostic categories in the dataset and offers insight into the study population. Notably, lung cancer (C34) is the most prevalent diagnosis.

Characteristic	Female			Male		
Number of patients (%)	3,491 (40)			5,269 (60)		
Median age ([IQR] *in years*)	63 [54-71]			65 [56-73]		
Median survival time ([IQR] *in months*)	14 [6-35]			14 [5-32]		
Deceased patients as of 2022-02-16 (%)	1,322 (38)			2,335 (44)		
Gastrointestinal (%) [C15-C18, C20, C25]	468 (14)			683 (13)		
Head+Neck (%) [C01-C14, C32]	102 (3)			242 (5)		
Liver (%) [C22]	218 (6)			575 (11)		
Lung (%) [C34]	1,400 (40)			2,048 (39)		
Sarcoma (%) [C40, C41, C46, C49]	337 (10)			417 (8)		
Skin (%) [C43, C44]	234 (7)			334 (6)		
Urogenital (%) [C61, C62, C64, C67]	64 (2)			507 (10)		
Other (%) [Supplementary Table 5]	668 (19)			463 (9)		
Median Sarcopenia Index ([IQR])	1.56 [1.38-1.75]			1.71 [1.47-1.99]		
**M Status**	**M0**	**M1**	**Mx**	**M0**	**M1**	**Mx**
Total (%)	1,518 (43)	1,060 (30)	913 (26)	2,573 (49)	1,553 (29)	1,143 (22)
Median age ([IQR] *in years*)	64 [54-71]	62 [54-70]	62 [52-72]	65 [56-73]	63 [56-71]	65 [57-73]
Median survival time ([IQR] *in months*)	18 [7-41]	11 [4-24]	15 [6-42]	17 [7-39]	10 [4-21]	15 [5-39]
Deceased patients as of 2022-02-16 (%)	448 (30)	587 (55)	287 (31)	932 (36)	893 (58)	510 (47)
Gastrointestinal (%) [C15-C18, C20, C25]	150 (10)	191 (18)	127 (14)	263 (10)	254 (16)	166 (15)
Head+Neck (%) [C01-C14, C32]	75 (5)	6 (1)	21 (2)	185 (7)	22 (1)	35 (3)
Liver (%) [C22]	29 (2)	58 (6)	131 (14)	96 (4)	98 (6)	381 (33)

Lung (%) [C34]	775 (51)	509 (48)	116 (13)	1,173 (46)	727 (47)	148 (13)
Sarcoma (%) [C40, C41, C46, C49]	188 (12)	76 (7)	73 (8)	220 (9)	99 (6)	98 (9)
Skin (%) [C43, C44]	174 (12)	23 (2)	37 (4)	247 (10)	42 (3)	45 (4)
Urogenital (%) [C61, C62, C64, C67]	15 (1)	17 (2)	32 (4)	232 (9)	162 (13)	113 (10)
Other (%) [Supplementary Table 5]	122 (7)	180 (17)	376 (41)	157 (6)	149 (10)	157 (14)
Median Sarcopenia Index ([IQR])	1.56 [1.38-1.77]	1.54 [1.36-1.71]	1.59 [1.39-1.78]	1.73 [1.49-2.02]	1.68 [1.45-1.94]	1.70 [1.47-1.98]

Supplementary Table 3. Patient characteristics of the internal thorax cohort: The table presents the descriptive characteristics of patients within the internal thorax cohort, stratified by sex and metastatic status (M status). Patients are categorized into three groups based on their metastatic status: non-metastatic (M0), metastatic (M1), or unknown (Mx).

**Characteristic**	**Female**		**Male**	
Number of patients (%)	178 (40)		261 (60)	
Median age ([IQR] *in years*)	67 [57-75]		66 [56-74]	
Median survival time ([IQR] *in months*)	27 [10-61]		30 [12-54]	
Deceased patients as of 2022-02-16 (%)	88 (49)		109 (42)	
Head+Neck (%) [C01-C14, C32]	7 (4)		25 (10)	
Liver (%) [C22]	32 (18)		38 (15)	
Lung (%) [C34]	101 (57)		104 (40)	
Sarcoma (%) [C40, C41, C46, C49]	23 (13)		35 (13)	
Skin (%) [C43, C44]	15 (8)		9 (4)	
Urogenital (%) [C61, C62, C64, C67]	-		50 (19)	
Median Sarcopenia Index ([IQR])	2.22 [2.03-2.42]		2.52 [2.26-2.79]	
**M Status**	**M0**	**M1**	**M0**	**M1**
Total (%)	115 (65)	63 (35)	204 (78)	57 (21)
Median age ([IQR] *in years*)	67 [59-75]	66 [55-73]	66 [56-74]	67 [59-74]
Median survival time ([IQR] *in months*)	39 [13-83]	16 [7-27]	35 [16-67]	12 [3-24]
Deceased patients as of 2022-02-16 (%)	49 (43)	39 (62)	71 (35)	38 (67)
Head+Neck (%) [C01-C14, C32]	5 (4)	2 (3)	19 (9)	5 (4)
Liver (%) [C22]	16 (14)	16 (25)	23 (11)	16 (13)
Lung (%) [C34]	66 (57)	35 (56)	74 (36)	66 (57)
Sarcoma (%) [C40, C41, C46, C49]	23 (20)	-	34 (17)	23 (40)
Skin (%) [C43, C44]	5 (4)	10 (16)	4 (2)	5 (9)
Urogenital (%) [C61, C62, C64, C67]	-	-	50 (25)	2 (4)
Median Sarcopenia Index ([IQR])	2.22 [2.01-2.44]	2.20 [2.06-2.42]	2.54 [2.29-2.82]	2.48 [2.23-2.66]

Supplementary Table 4. Patient characteristics of the external validation cohort: This table provides the descriptive characteristics of patients in the external validation cohort, stratified by sex and metastatic status (M status). Patients are classified as non-metastatic (M0) or metastatic (M1).

ICD-10-GM-Code	ICD-Description	Total (Female %)	Age (median [IQR])
C80	Malignant neoplasm, without specification of site	263 (44)	65 [15]
C50	Malignant neoplasm of breast	261 (98)	59 [21]
C45	A benign or malignant tumor affecting the lining of the chest or abdomen.	96 (14)	74 [13]
C48	Malignant neoplasm of retroperitoneum and peritoneum parietal, pelvic	68 (38)	59 [17]
C79	Secondary malignant neoplasm of other and unspecified sites	62 (57)	68 [16]
C23	Malignant neoplasm of gallbladder	47 (58)	69 [20]
C74	Malignant neoplasm of adrenal gland	38 (61)	59 [22]
C69	Malignant neoplasm of eye and adnexa	37 (51)	70 [22]
C56	Malignant neoplasm of ovary	36 (100)	62 [17]
C53	Malignant neoplasm of cervix uteri	36 (36)	57 [14]
C21	Malignant neoplasm of anus and anal canal	35 (57)	59 [17]
C54	Malignant neoplasm of corpus uteri	34 (100)	56 [13]
C38	Malignant neoplasm of heart, mediastinum and pleura	30 (37)	66 [19]
C31	Malignant neoplasm of accessory sinuses	26 (50)	61 [17]
C26	Malignant neoplasm of other and ill-defined digestive organs	24 (75)	68 [20]
C75	Malignant neoplasm of other endocrine glands and related structures	23 (61)	59 [15]
C37	Malignant neoplasm of thymus	22 (46)	61 [25]
C65	Malignant neoplasm of renal pelvis	19 (53)	70 [10]
C60	Malignant neoplasm of penis	18 (0)	68 [13]
C51	Malignant neoplasm of vulva	16 (100)	72 [21]
C30	Malignant neoplasm of nasal cavity and middle ear	15 (40)	67 [25]
C78	Secondary malignant neoplasm of respiratory and digestive organs	14 (64)	70 [20]
C47	Malignant neoplasm of peripheral nerves and autonomic nervous system	14 (43)	55 [41]
C76	Malignant neoplasm of other and ill-defined sites	12 (42)	72 [17]
C55	Malignant neoplasm of uterus, part unspecified	12 (100)	64 [23]
C72	Malignant neoplasm of spinal cord, cranial nerves and other parts of central nervous system	11 (18)	37 [32]
C19	Malignant neoplasm of rectosigmoid junction	10 (30)	63 [10]
C68	Malignant neoplasm of other and unspecified urinary organs	9 (11)	73 [12]

C63	Malignant neoplasm of other and unspecified male genital organs	7 (0)	57 [32]
C14	Malignant neoplasm of other and ill-defined sites in the lip, oral cavity, and pharynx	7 (43)	66 [13]
C57	Malignant neoplasm of other and unspecified female genital organs	6 (100)	58 [11]
C33	Malignant neoplasm of trachea	6 (17)	66 [3]
C66	Malignant neoplasm of ureter	5 (20)	75 [20]
C58	Malignant neoplasm of placenta	3 (100)	35 [2]
C39	Malignant neoplasm of other and ill-defined sites in the respiratory system and intrathoracic organs	2 (0)	63 [2]
C77	Secondary and unspecified malignant neoplasm of lymph nodes	1 (0)	56 [0]
C70	Malignant neoplasm of meninges	1 (100)	55 [0]

Supplementary Table 5. ICD-10 codes included in the cancer group "Other": This table presents all ICD-10-GM codes categorized under the "Other" cancer group in the internal cohort. These codes represent rare malignant tumors that could not be assigned to any of the major cancer groups. The table is sorted in descending order by frequency of occurrence. This grouping was used to ensure that patients with less frequent or rare solid tumor diagnoses were retained in the analysis.

CT Region	Processing time in seconds
Whole Body	150
Abdomen	49
Thorax	45

Supplementary Table 6. Processing times of the body composition analysis (BCA) network across different CT scan types: This table reports the average processing times required by the BCA network to analyze CT scans of varying anatomical coverage, including whole-body, abdomen, and thorax scans. The benchmarks were obtained using an Nvidia Titan RTX GPU with 24 GB of RAM. Times reflect the end-to-end computation per scan, including tissue segmentation and feature extraction.

Cancer Group	N	P	Estimate (95% CI)
All Patients	10340	P < 0.001	0.56 (0.52 to 0.59)
Lung	3642	P < 0.001	0.72 (0.65 to 0.80)
Git	1490	P < 0.001	0.66 (0.57 to 0.77)
Other	1326	P < 0.001	0.54 (0.44 to 0.66)
Liver	1162	P < 0.001	0.64 (0.54 to 0.77)
Sarcoma	936	P < 0.001	0.66 (0.52 to 0.83)
Urogenital	786	P < 0.001	0.52 (0.41 to 0.65)
Skin	624	P < 0.001	0.63 (0.48 to 0.81)
Headneck	374	P < 0.001	0.28 (0.19 to 0.39)

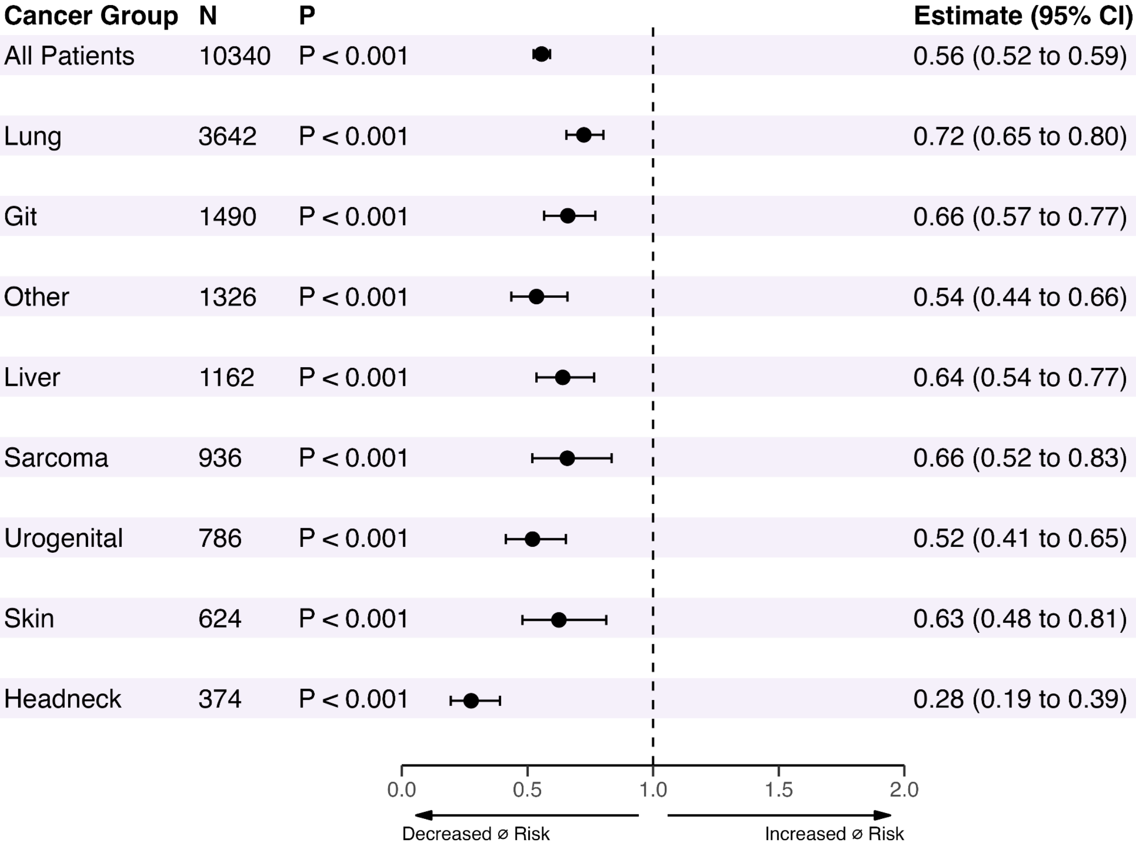

Supplementary Figure 1. Effect of the Sarcopenia Index from multivariable Cox regression in the internal abdomen cohort: This forest plot summarizes the results of a multivariable Cox proportional hazards regression analysis (using robust standard errors) conducted on the internal abdomen cohort. The analysis includes hazard ratio (HR) estimates, 95% confidence intervals (CIs), P-values, and total case counts, shown both for the overall cohort and stratified by cancer group. Covariates in the model included age at CT, sex, metastatic status, and cancer group. Importantly, the dataset did not meet the proportional hazards assumption; thus, the reported HR for the Sarcopenia Index should be interpreted as a time-averaged effect rather than a constant over time.

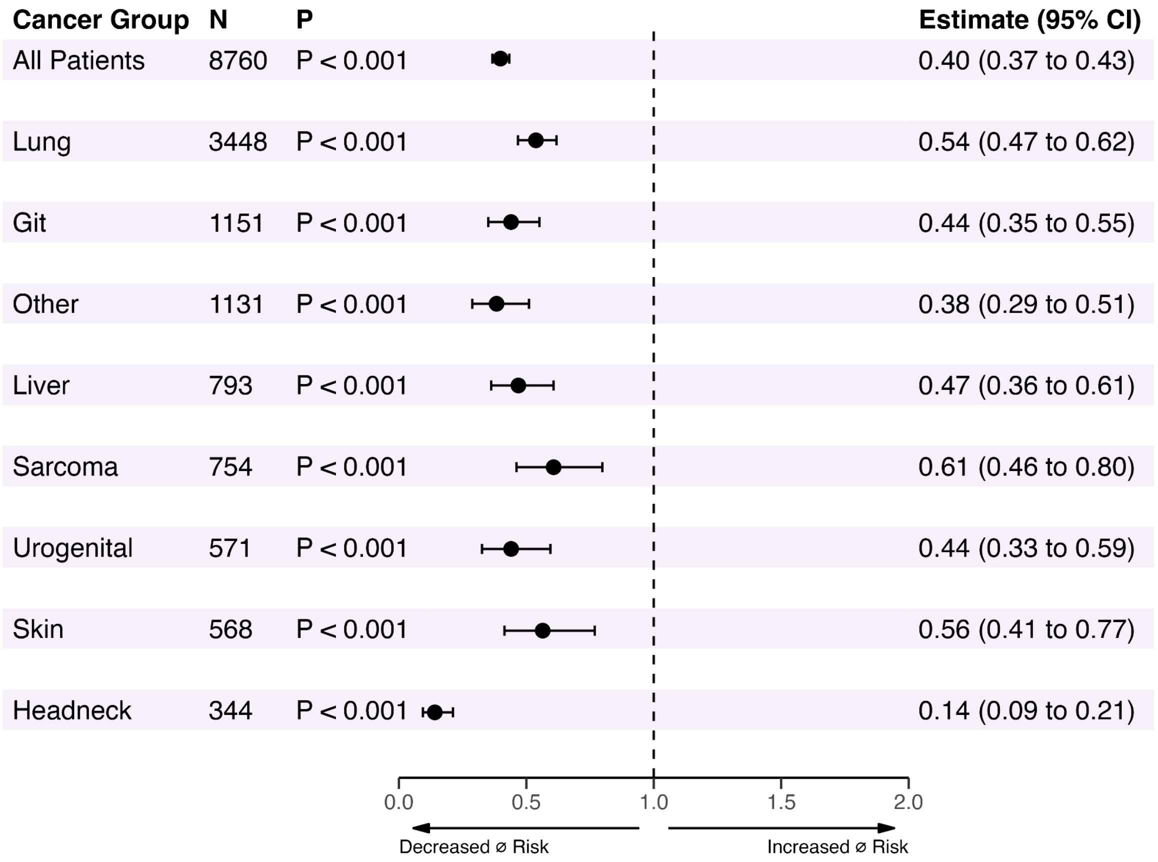

Supplementary Figure 2. Effect of the Sarcopenia Index from multivariable Cox regression in the internal thorax cohort: This forest plot summarizes the results of a multivariable Cox proportional hazards regression analysis (using robust standard errors) conducted on the internal thorax cohort. The analysis includes hazard ratio (HR) estimates, 95% confidence intervals (CIs), P-values, and total case counts, shown both for the overall cohort and stratified by cancer group. Covariates in the model included age at CT, sex, metastatic status, and cancer group. Importantly, the dataset did not meet the proportional hazards assumption; thus, the reported HR for the Sarcopenia Index should be interpreted as a time-averaged effect rather than a constant over time.

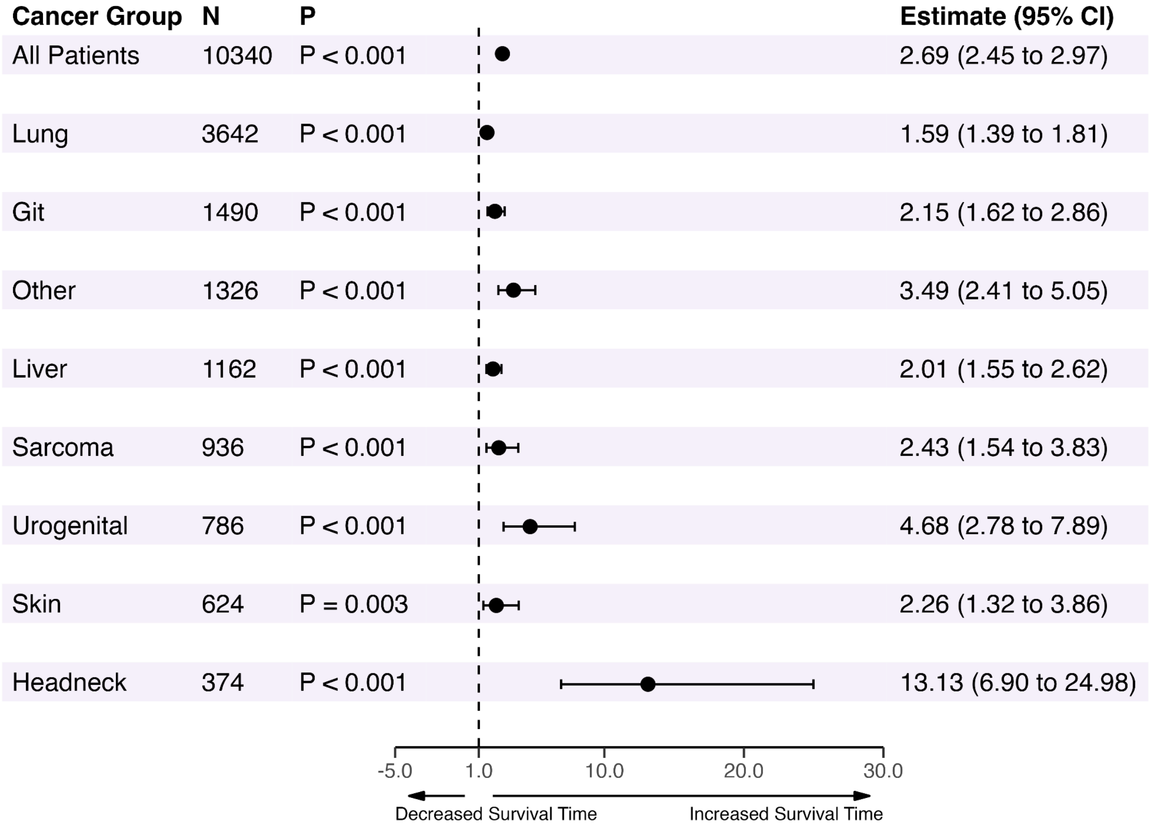

Supplementary Figure 3. Effect of the Sarcopenia Index from multivariable Accelerated Failure Time (AFT) model in the internal abdomen cohort: This forest plot shows the results of a multivariable Accelerated Failure Time (AFT) model applied to the internal abdomen cohort, evaluating the effect of the Sarcopenia Index on survival time. The model included covariates for age at CT, sex, metastatic status, and cancer group. The plot shows the total number of cases, estimated time ratios, 95% confidence intervals (CIs), and corresponding P-values for both the overall cohort and individual cancer groups.

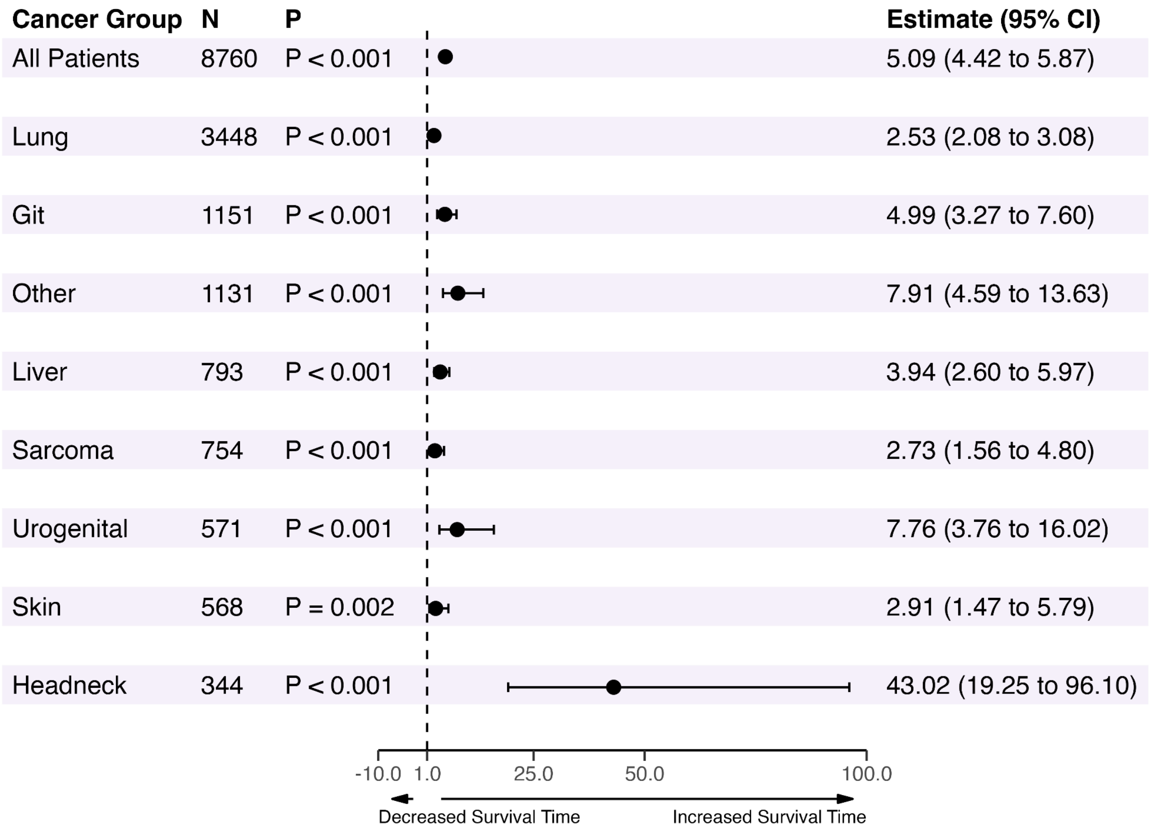

Supplementary Figure 4. Effect of the Sarcopenia Index from multivariable Accelerated Failure Time (AFT) model in the internal thorax cohort: This forest plot presents the results of a multivariable Accelerated Failure Time (AFT) model applied to the internal thorax cohort, evaluating the effect of the Sarcopenia Index on survival time. The model included covariates for age at CT, sex, metastatic status, and cancer group. The plot shows the total number of cases, estimated time ratios, 95% confidence intervals (CIs), and corresponding P-values for both the overall cohort and individual cancer groups.

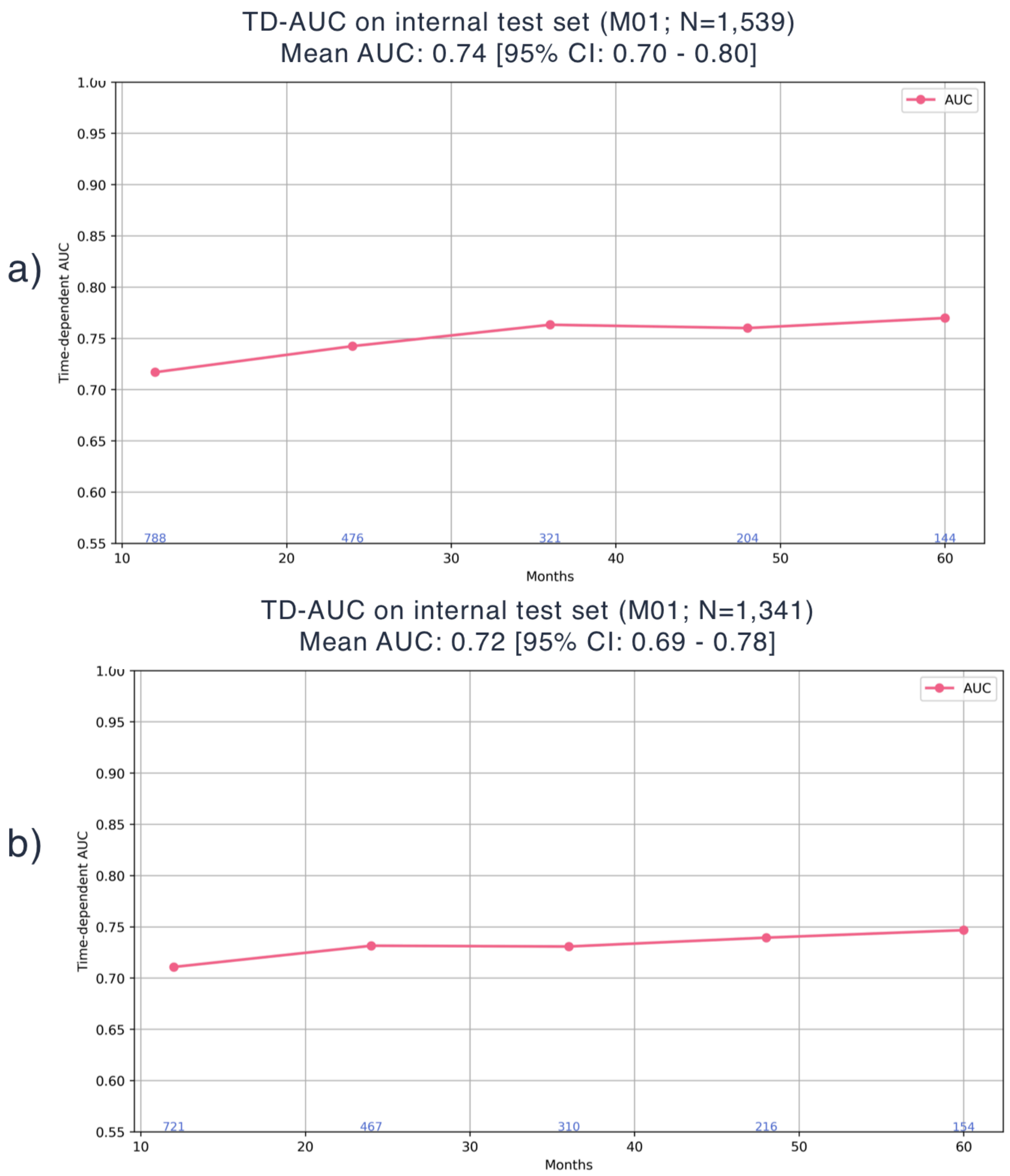

Supplementary Figure 5. Time-dependent AUC for internal abdomen and thorax models with non-metastatic and metastatic patients (M0 and M1): The figure shows time-dependent Area Under the Curve (AUC) values for models trained on the internal abdomen and thorax cohorts, with combined metastatic status (non-metastatic [M0] and metastatic [M1]). Panel (a) shows the AUC values over time for the abdomen-based model, while panel (b) displays the corresponding results for the thorax-based model. Purple numbers along the x-axis represent the number of patients at risk at each evaluation time point.

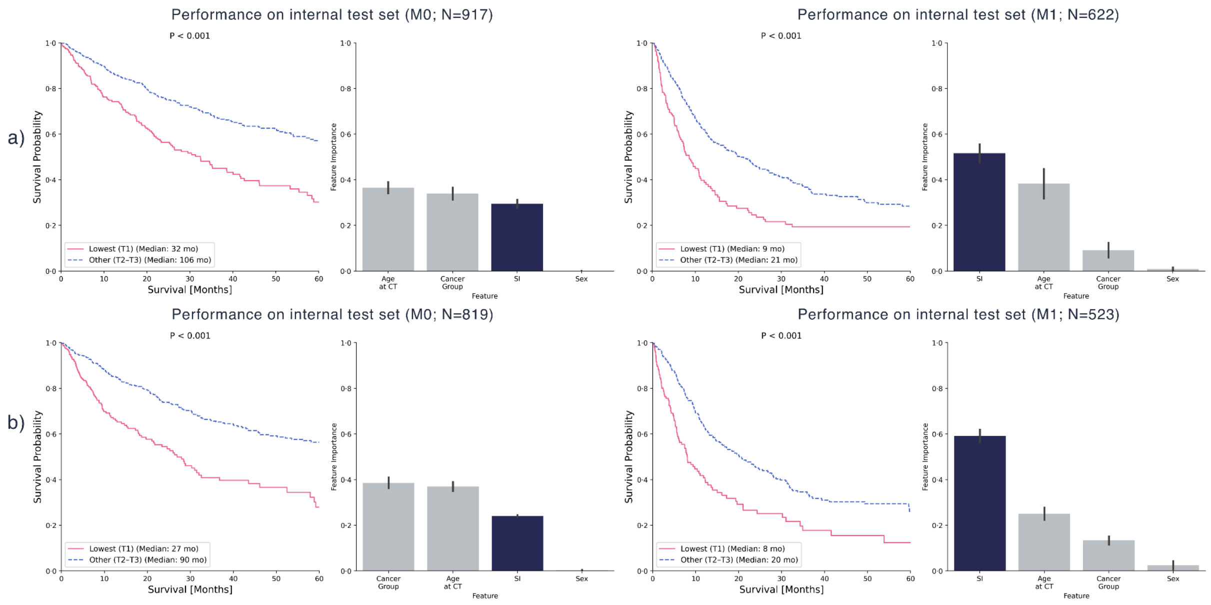

Supplementary Figure 6. Machine learning results for distinct metastasis groups based on the internal abdomen and thorax cohorts for non-metastatic (M0) and metastatic (M1) models: This figure presents results from gradient-boosted survival tree models trained separately for non-metastatic (M0) and metastatic (M1) patients within the internal abdomen and thorax cohorts. Kaplan-Meier survival curves compare the lowest tertile (T1) with the combined upper tertiles (T2-T3) of predicted survival times. The curves include annotated median survival times (in months) for each group. Panel (a) shows results for the internal abdomen test cohort, with separate plots for M0 (left) and M1 (right) patients. Panel (b) displays corresponding results for the internal thorax test cohort, again stratified by metastatic status (M0, left; M1, right). Notably, SI appears to be particularly important for survival prediction in patients with progressed disease (M1).

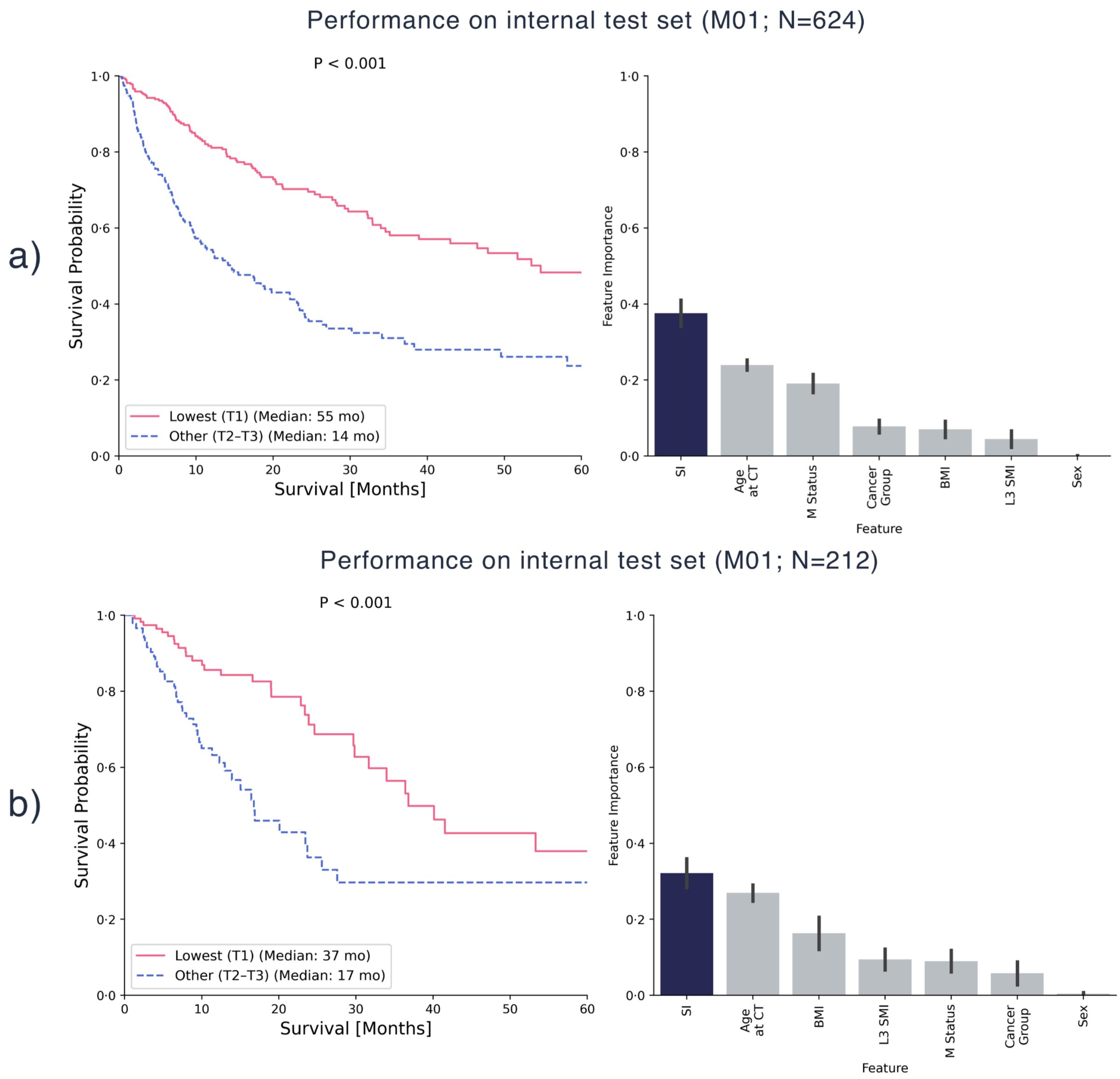

Supplementary Figure 7. Machine learning results for SI, L3-SMI, and BMI comparison: This figure presents machine learning results from models trained with multiple body composition features (Sarcopenia Index [SI], L3-Skeletal Muscle Index [L3 SMI], and Body Mass Index [BMI]) within the internal cohorts. Kaplan-Meier survival curves compare the lowest tertile (T1) with the combined upper tertiles (T2-T3) of survival times predicted by gradient-boosted survival trees. The curves include annotated median survival times (in months) for each group. Panel (a) shows results for the internal abdomen test cohort, while panel (b) shows results for the internal thorax test cohort. Across both cohorts, the SI consistently emerged as the most important feature for predicting survival.

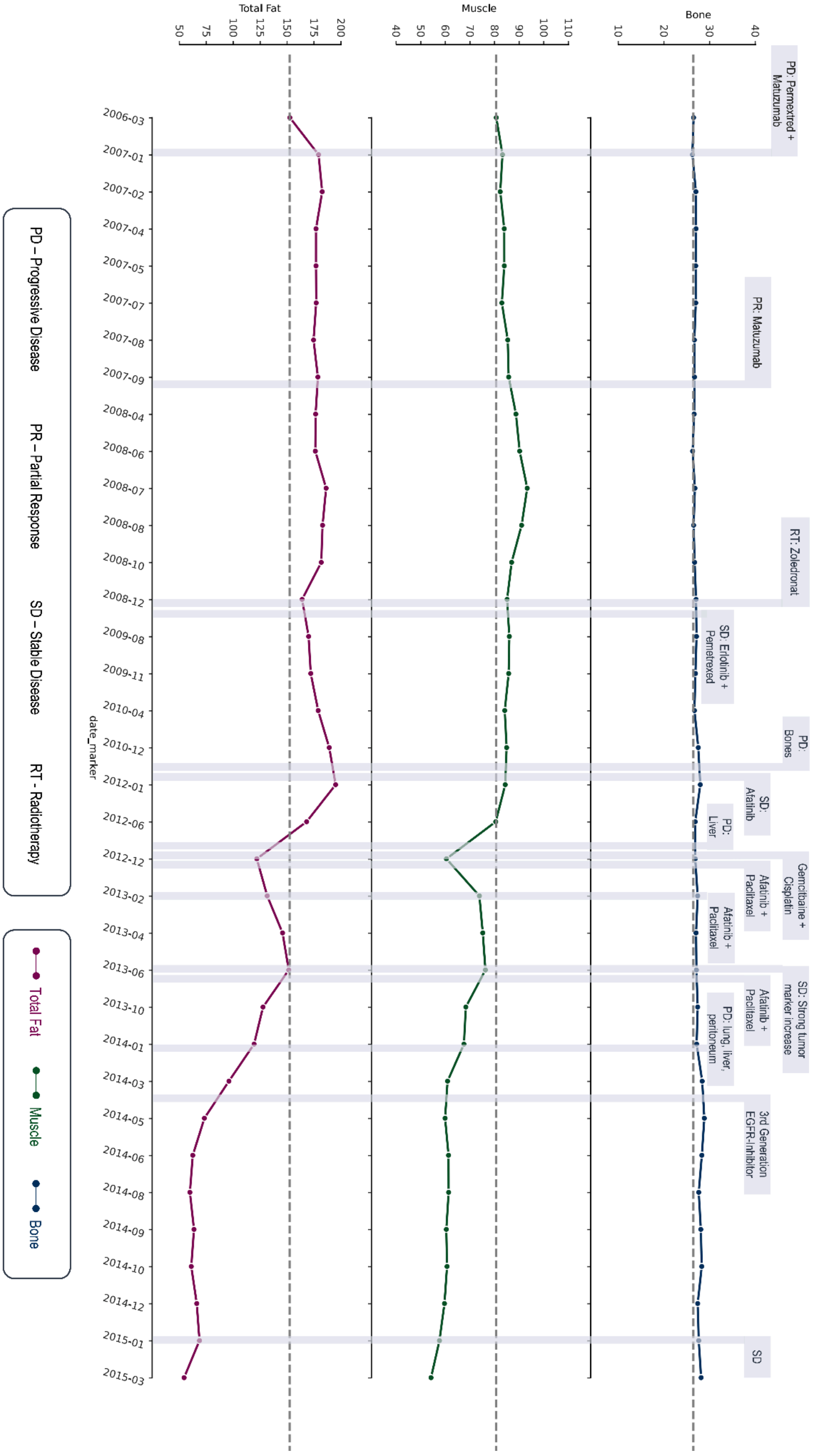

Supplementary Figure 8. Longitudinal body composition changes in a lung cancer patient with osteoblastic metastasis: The figure displays a longitudinal analysis of body composition features in a lung cancer patient diagnosed with osteoblastic metastasis (first detected in 2009), based on 34

follow-up thorax CT scans. The line plots depict the temporal evolution of the BCA features, muscle, bone, and total fat: bone volume remains relatively stable, while muscle and total adipose tissue volumes show a progressive decline over time. These trends may indicate a worsening clinical condition, as the patient passed away shortly after the final follow-up scan. Relevant treatment events are annotated along the timeline.

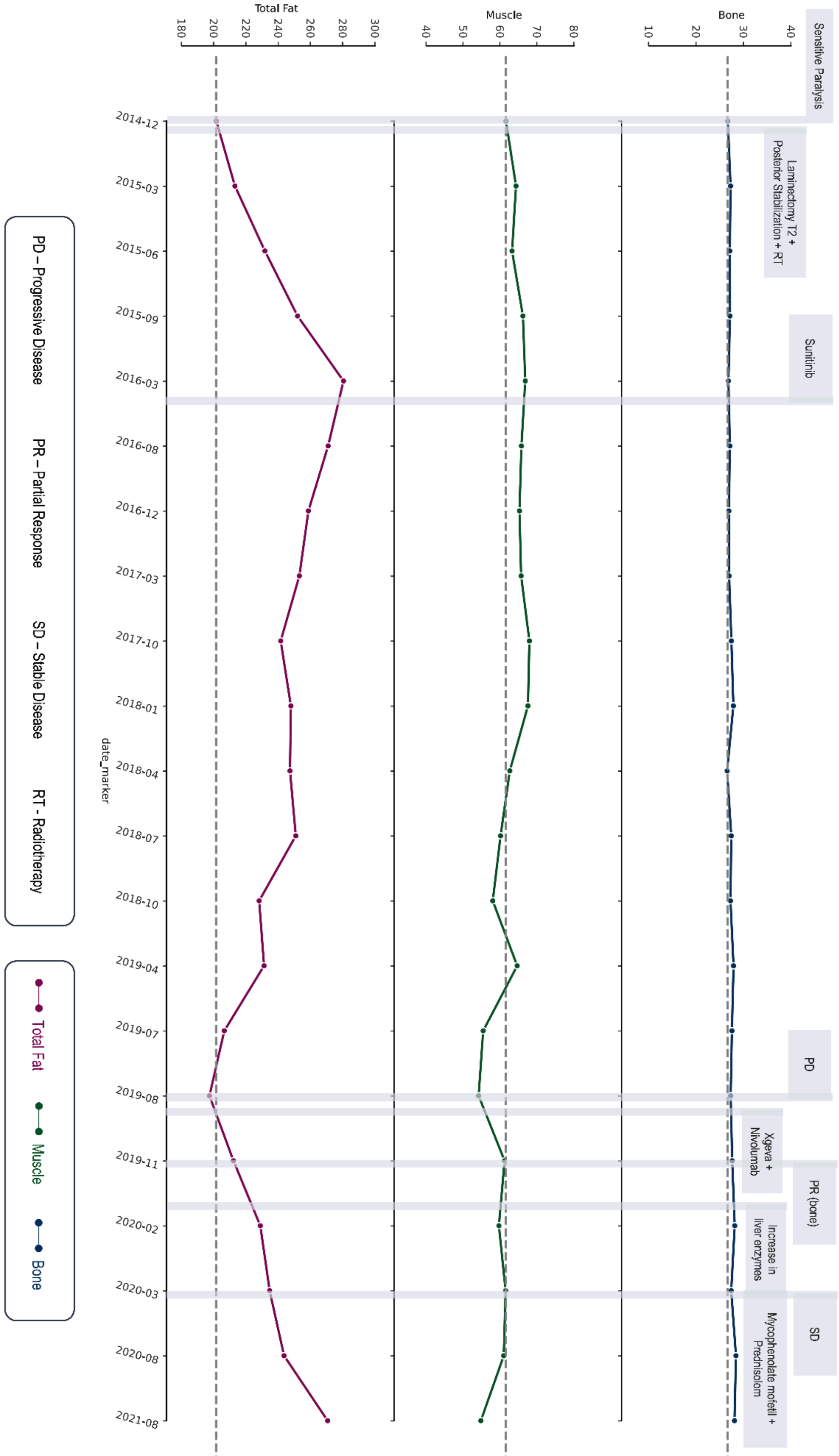

Supplementary Figure 9. Longitudinal body composition changes in a patient with osteolytic metastasis: This figure illustrates the longitudinal development of body composition features in a

patient diagnosed with osteolytic metastasis (first detected in 2014), based on 20 follow-up thorax CT scans. The line plots show that bone volume remains relatively stable over time, while total adipose tissue volume exhibits noticeable fluctuations throughout the course of the disease. These variations may reflect changes in the patient's physiological state or treatment response.

Body Composition Analysis Report

Summary

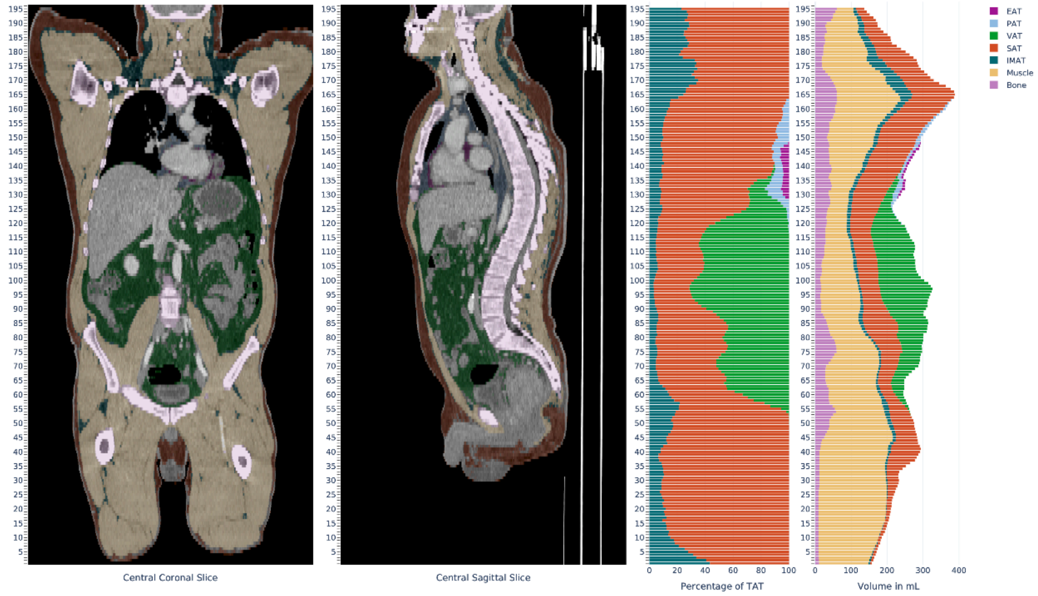

Equidistant Axial Slices

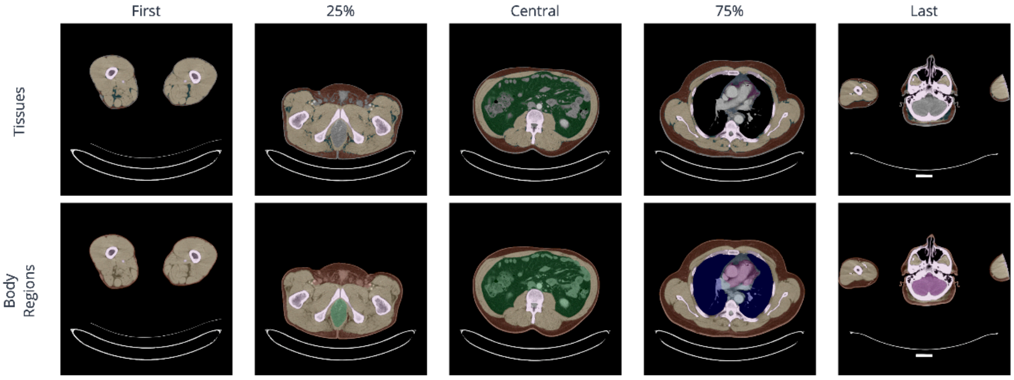

Tissue Heatmaps

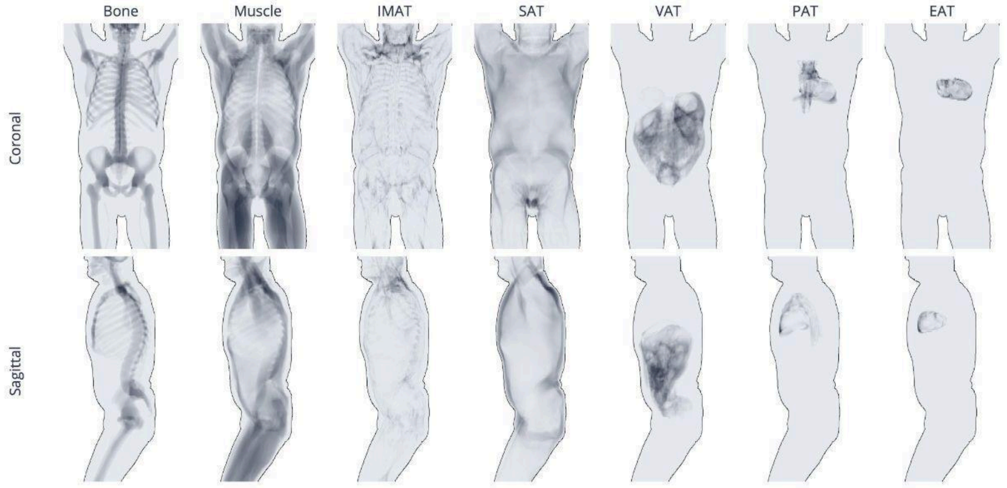

Aggregated Measurements

Whole Scan

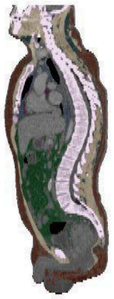

All Voxels

	Bone	Muscle	TAT	IMAT	SAT	VAT	PAT	EAT
**Mean**	30.04 mL	125.86 mL	106.89 mL	11.68 mL	62.56 mL	30.06 mL	1.95 mL	0.64 mL
**StdDev**	15.31 mL	42.95 mL	57.14 mL	10.01 mL	32.30 mL	43.95 mL	4.13 mL	2.00 mL
**Minimum**	9.12 mL	45.85 mL	9.19 mL	0.99 mL	5.11 mL	0.00 mL	0.00 mL	0.00 mL
**25%**	15.88 mL	91.74 mL	67.17 mL	5.90 mL	39.97 mL	0.00 mL	0.00 mL	0.00 mL
**Median**	29.26 mL	130.60 mL	125.56 mL	9.56 mL	57.92 mL	0.00 mL	0.00 mL	0.00 mL
**75%**	39.94 mL	161.84 mL	145.45 mL	12.74 mL	87.95 mL	57.03 mL	0.00 mL	0.00 mL
**Maximum**	61.21 mL	196.80 mL	205.61 mL	49.08 mL	120.64 mL	146.68 mL	16.99 mL	8.90 mL
**Total**	5.888 L	24.669 L	20.951 L	2.289 L	12.263 L	5.891 L	381.26 mL	126.10 mL
**MeanHU**	310.96 HU	38.78 HU	-88.38 HU	-63.70 HU	-92.61 HU	-89.54 HU	-88.18 HU	-71.78 HU

Without Limbs

	Bone	Muscle	TAT	IMAT	SAT	VAT	PAT	EAT
**Mean**	25.75 mL	76.79 mL	95.46 mL	10.05 mL	52.76 mL	30.06 mL	1.95 mL	0.64 mL
**StdDev**	17.83 mL	52.86 mL	64.60 mL	9.09 mL	35.50 mL	43.95 mL	4.13 mL	2.00 mL
**Minimum**	0.00 mL	0.00 mL	0.00 mL	0.00 mL	0.00 mL	0.00 mL	0.00 mL	0.00 mL
**25%**	12.32 mL	44.34 mL	30.54 mL	5.13 mL	21.21 mL	0.00 mL	0.00 mL	0.00 mL
**Median**	28.76 mL	81.22 mL	112.62 mL	9.32 mL	55.09 mL	0.00 mL	0.00 mL	0.00 mL
**75%**	39.30 mL	118.02 mL	142.27 mL	12.37 mL	77.97 mL	57.03 mL	0.00 mL	0.00 mL
**Maximum**	59.29 mL	184.63 mL	204.92 mL	42.79 mL	119.85 mL	146.68 mL	16.99 mL	8.90 mL
**Total**	5.048 L	15.051 L	18.711 L	1.971 L	10.342 L	5.891 L	381.26 mL	126.10 mL
**MeanHU**	271.34 HU	35.15 HU	-88.86 HU	-64.36 HU	-93.37 HU	-89.54 HU	-88.18 HU	-71.78 HU

Abdominal Cavity

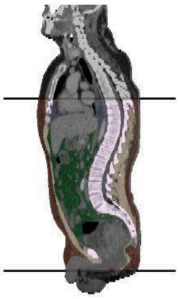

All Voxels

	Bone	Muscle	TAT	IMAT	SAT	VAT	PAT	EAT
Mean	32.96 mL	105.63 mL	134.89 mL	9.17 mL	63.64 mL	59.51 mL	1.82 mL	0.74 mL
StdDev	12.06 mL	38.78 mL	44.37 mL	2.42 mL	17.99 mL	45.54 mL	4.27 mL	2.05 mL
Minimum	13.77 mL	49.60 mL	65.68 mL	4.81 mL	33.92 mL	0.00 mL	0.00 mL	0.00 mL
25%	24.76 mL	74.62 mL	88.11 mL	7.24 mL	52.87 mL	17.97 mL	0.00 mL	0.00 mL
Median	32.32 mL	101.59 mL	139.11 mL	9.26 mL	57.67 mL	56.80 mL	0.00 mL	0.00 mL
75%	40.65 mL	137.10 mL	172.99 mL	10.86 mL	74.31 mL	104.42 mL	0.00 mL	0.00 mL
Maximum	59.29 mL	196.80 mL	205.61 mL	15.06 mL	100.48 mL	146.68 mL	16.99 mL	8.90 mL
Total	3.263 L	10.457 L	13.354 L	908.28 mL	6.301 L	5.891 L	180.52 mL	73.21 mL
MeanHU	261.40 HU	35.26 HU	-88.04 HU	-55.49 HU	-91.39 HU	-89.54 HU	-91.29 HU	-73.69 HU

Without Limbs

	Bone	Muscle	TAT	IMAT	SAT	VAT	PAT	EAT
Mean	32.49 mL	100.14 mL	132.34 mL	8.91 mL	61.35 mL	59.51 mL	1.82 mL	0.74 mL
StdDev	12.97 mL	38.00 mL	47.83 mL	2.80 mL	19.44 mL	45.54 mL	4.27 mL	2.05 mL
Minimum	0.00 mL	0.00 mL	14.73 mL	0.00 mL	14.73 mL	0.00 mL	0.00 mL	0.00 mL
25%	24.60 mL	68.95 mL	85.00 mL	6.88 mL	51.80 mL	17.97 mL	0.00 mL	0.00 mL
Median	32.32 mL	98.92 mL	138.24 mL	9.22 mL	56.69 mL	56.80 mL	0.00 mL	0.00 mL
75%	40.65 mL	135.25 mL	172.32 mL	10.86 mL	72.30 mL	104.42 mL	0.00 mL	0.00 mL
Maximum	59.29 mL	184.63 mL	204.92 mL	15.06 mL	99.48 mL	146.68 mL	16.99 mL	8.90 mL
Total	3.216 L	9.914 L	13.102 L	882.33 mL	6.074 L	5.891 L	180.52 mL	73.21 mL
MeanHU	258.73 HU	35.11 HU	-88.14 HU	-55.63 HU	-91.58 HU	-89.54 HU	-91.29 HU	-73.69 HU

Thoracic Cavity

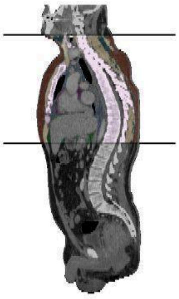

All Voxels

	Bone	Muscle	TAT	IMAT	SAT	VAT	PAT	EAT
Mean	40.68 mL	108.06 mL	139.60 mL	19.26 mL	96.69 mL	15.47 mL	6.15 mL	2.03 mL
StdDev	10.53 mL	42.75 mL	11.79 mL	12.87 mL	18.69 mL	28.58 mL	5.31 mL	3.15 mL
Minimum	17.93 mL	49.60 mL	121.91 mL	8.63 mL	55.65 mL	0.00 mL	0.00 mL	0.00 mL
25%	34.82 mL	58.74 mL	130.79 mL	10.84 mL	84.84 mL	0.00 mL	0.32 mL	0.00 mL
Median	39.36 mL	117.42 mL	138.56 mL	12.56 mL	100.03 mL	0.00 mL	6.75 mL	0.00 mL
75%	45.42 mL	146.79 mL	144.86 mL	21.06 mL	112.07 mL	19.60 mL	10.71 mL	5.26 mL
Maximum	61.21 mL	176.01 mL	170.52 mL	49.08 mL	120.64 mL	105.70 mL	16.99 mL	8.90 mL
Total	2.522 L	6.699 L	8.655 L	1.194 L	5.995 L	959.00 mL	381.26 mL	126.10 mL
MeanHU	229.77 HU	31.18 HU	-92.24 HU	-67.47 HU	-98.53 HU	-88.06 HU	-88.18 HU	-71.78 HU

Without Limbs

	Bone	Muscle	TAT	IMAT	SAT	VAT	PAT	EAT
Mean	36.95 mL	93.01 mL	130.78 mL	17.76 mL	89.37 mL	15.47 mL	6.15 mL	2.03 mL
StdDev	10.32 mL	35.03 mL	17.15 mL	10.35 mL	19.80 mL	28.58 mL	5.31 mL	3.15 mL
Minimum	10.01 mL	49.60 mL	90.55 mL	8.63 mL	54.76 mL	0.00 mL	0.00 mL	0.00 mL
25%	32.11 mL	58.06 mL	124.97 mL	10.84 mL	72.12 mL	0.00 mL	0.32 mL	0.00 mL
Median	38.65 mL	89.97 mL	131.24 mL	12.56 mL	92.61 mL	0.00 mL	6.75 mL	0.00 mL
75%	42.32 mL	126.60 mL	141.79 mL	19.21 mL	104.88 mL	19.60 mL	10.71 mL	5.26 mL
Maximum	55.71 mL	163.00 mL	169.63 mL	42.79 mL	119.85 mL	105.70 mL	16.99 mL	8.90 mL
Total	2.291 L	5.766 L	8.108 L	1.101 L	5.541 L	959.00 mL	381.26 mL	126.10 mL
MeanHU	229.59 HU	31.56 HU	-91.50 HU	-67.60 HU	-97.53 HU	-88.06 HU	-88.18 HU	-71.78 HU

Mediastinum

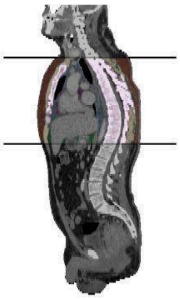

All Voxels

	Bone	Muscle	TAT	IMAT	SAT	VAT	PAT	EAT
Mean	40.96 mL	97.12 mL	139.58 mL	13.01 mL	96.65 mL	19.57 mL	7.78 mL	2.57 mL
StdDev	8.30 mL	41.25 mL	10.92 mL	4.12 mL	19.72 mL	30.92 mL	4.79 mL	3.34 mL
Minimum	28.73 mL	49.60 mL	124.85 mL	8.63 mL	55.65 mL	0.00 mL	0.01 mL	0.00 mL
25%	35.90 mL	57.03 mL	131.34 mL	10.48 mL	84.89 mL	0.00 mL	3.81 mL	0.00 mL
Median	39.33 mL	87.34 mL	139.11 mL	11.94 mL	100.48 mL	1.44 mL	7.45 mL	0.00 mL
75%	44.36 mL	130.96 mL	144.38 mL	13.47 mL	112.51 mL	25.88 mL	11.56 mL	5.88 mL
Maximum	61.21 mL	176.01 mL	170.52 mL	31.09 mL	120.64 mL	105.70 mL	16.99 mL	8.90 mL
Total	2.007 L	4.759 L	6.839 L	637.27 mL	4.736 L	959.00 mL	381.26 mL	126.10 mL
MeanHU	221.24 HU	32.00 HU	-92.74 HU	-60.22 HU	-99.00 HU	-88.06 HU	-88.18 HU	-71.78 HU

Without Limbs

	Bone	Muscle	TAT	IMAT	SAT	VAT	PAT	EAT
Mean	40.22 mL	94.67 mL	136.73 mL	12.78 mL	94.02 mL	19.57 mL	7.78 mL	2.57 mL
StdDev	6.95 mL	37.40 mL	12.10 mL	3.52 mL	18.68 mL	30.92 mL	4.79 mL	3.34 mL
Minimum	28.73 mL	49.60 mL	113.40 mL	8.63 mL	54.76 mL	0.00 mL	0.01 mL	0.00 mL
25%	35.90 mL	57.03 mL	129.28 mL	10.48 mL	83.88 mL	0.00 mL	3.81 mL	0.00 mL
Median	39.33 mL	87.34 mL	134.17 mL	11.94 mL	99.10 mL	1.44 mL	7.45 mL	0.00 mL
75%	44.36 mL	130.96 mL	142.16 mL	13.47 mL	105.30 mL	25.88 mL	11.56 mL	5.88 mL
Maximum	55.71 mL	163.00 mL	169.63 mL	27.58 mL	119.85 mL	105.70 mL	16.99 mL	8.90 mL
Total	1.971 L	4.639 L	6.700 L	626.02 mL	4.607 L	959.00 mL	381.26 mL	126.10 mL
MeanHU	220.79 HU	32.05 HU	-92.46 HU	-60.17 HU	-98.69 HU	-88.06 HU	-88.18 HU	-71.78 HU

Pericardium

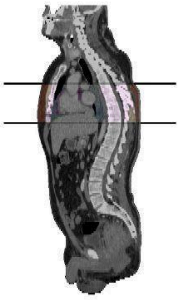

All Voxels

	Bone	Muscle	TAT	IMAT	SAT	VAT	PAT	EAT
Mean	40.60 mL	84.02 mL	135.12 mL	11.63 mL	99.41 mL	7.52 mL	10.81 mL	5.73 mL
StdDev	3.14 mL	24.90 mL	7.29 mL	1.04 mL	6.32 mL	9.39 mL	3.40 mL	2.57 mL
Minimum	35.90 mL	51.31 mL	124.85 mL	10.02 mL	84.89 mL	0.00 mL	6.69 mL	0.00 mL
25%	38.30 mL	58.97 mL	130.35 mL	10.83 mL	95.46 mL	0.00 mL	7.61 mL	5.22 mL
Median	39.40 mL	81.69 mL	132.76 mL	11.88 mL	100.25 mL	1.84 mL	10.80 mL	6.32 mL
75%	43.83 mL	101.58 mL	141.46 mL	12.33 mL	105.26 mL	16.79 mL	13.48 mL	7.48 mL
Maximum	46.67 mL	126.74 mL	150.43 mL	13.71 mL	106.71 mL	25.88 mL	16.99 mL	8.90 mL
Total	893.30 mL	1.848 L	2.973 L	255.91 mL	2.187 L	165.55 mL	237.89 mL	126.10 mL
MeanHU	211.14 HU	30.00 HU	-93.29 HU	-59.27 HU	-99.13 HU	-87.60 HU	-91.52 HU	-71.78 HU

Without Limbs

	Bone	Muscle	TAT	IMAT	SAT	VAT	PAT	EAT
Mean	40.60 mL	84.02 mL	134.23 mL	11.63 mL	98.53 mL	7.52 mL	10.81 mL	5.73 mL
StdDev	3.14 mL	24.90 mL	7.34 mL	1.04 mL	6.30 mL	9.39 mL	3.40 mL	2.57 mL
Minimum	35.90 mL	51.31 mL	124.05 mL	10.02 mL	83.88 mL	0.00 mL	6.69 mL	0.00 mL
25%	38.30 mL	58.97 mL	129.39 mL	10.83 mL	94.69 mL	0.00 mL	7.61 mL	5.22 mL
Median	39.40 mL	81.69 mL	131.87 mL	11.88 mL	99.36 mL	1.84 mL	10.80 mL	6.32 mL
75%	43.83 mL	101.58 mL	140.52 mL	12.33 mL	104.42 mL	16.79 mL	13.48 mL	7.48 mL
Maximum	46.67 mL	126.74 mL	149.58 mL	13.71 mL	105.76 mL	25.88 mL	16.99 mL	8.90 mL
Total	893.30 mL	1.848 L	2.953 L	255.91 mL	2.168 L	165.55 mL	237.89 mL	126.10 mL
MeanHU	211.14 HU	30.00 HU	-93.16 HU	-59.27 HU	-99.02 HU	-87.60 HU	-91.52 HU	-71.78 HU

Supplementary Figure 10. Example report from the body composition analysis (BCA) network for a whole-body CT scan: This figure shows an exemplary output report generated by the BCA network for a whole-body CT scan. The network segments various tissue types and automatically identifies anatomical regions within the scan. The report aggregates features both including and excluding the extremities for each potential body region: the whole scan, abdominal cavity, thoracic cavity, mediastinum, and pericardium. This output provides structured, region-specific measurements that are used in downstream analysis, ensuring consistency in tissue quantification and anatomical localization.

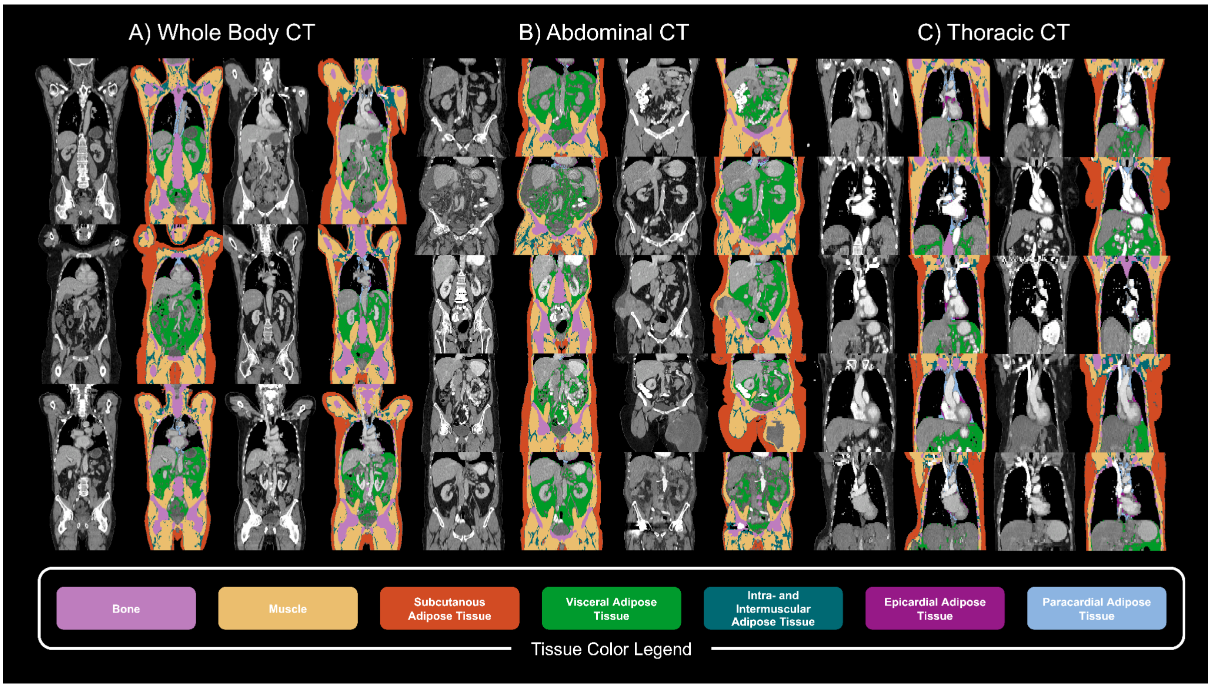

Supplementary Figure 11. Tissue segmentation examples from the body composition analysis (BCA) network: This figure illustrates segmentation outputs produced by the BCA network across different CT scan types. Panel A shows a whole-body CT scan, panel B an abdomen CT, and panel C a thorax CT. These examples reflect the range of input data used in the study and highlight the generalizability of the segmentation model across diverse body regions.

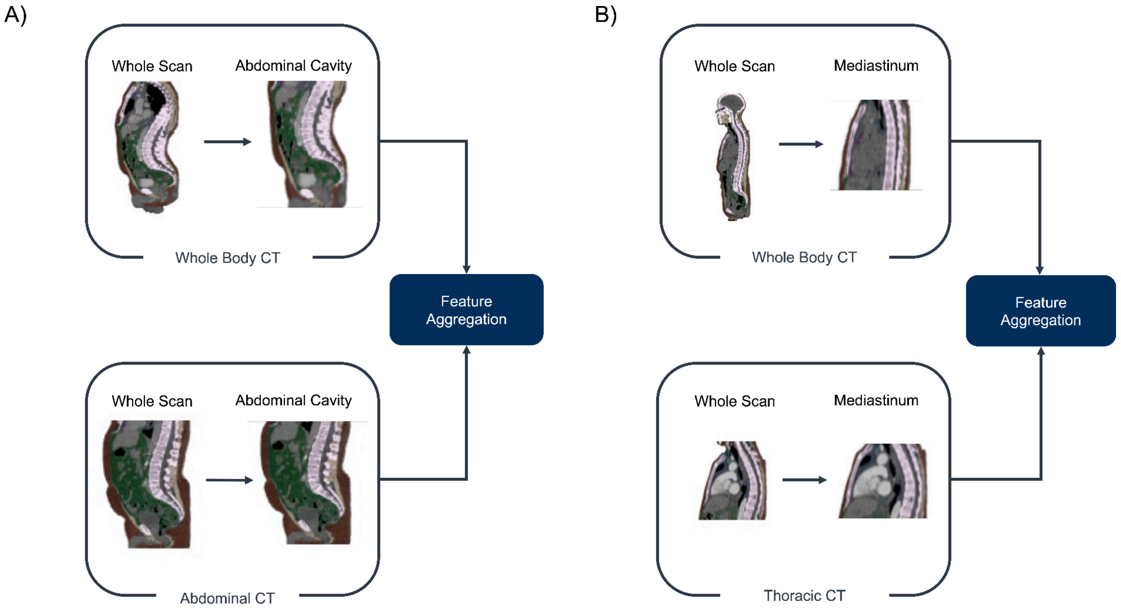

Supplementary Figure 12. Feature extraction using standardized body regions in body composition analysis (BCA): This figure illustrates the process of BCA feature extraction from specific anatomical regions identified automatically by the BCA network. Panel A shows feature extraction from the abdominal cavity, while panel B depicts the feature extraction from the mediastinum. Regardless of the CT scan's overall coverage, the network consistently detects predefined body regions and aggregates quantitative features from the segmented tissues within each region. This approach ensures the usage of a unified region across patients.
